# In Silico Identification and In Vitro Evaluation of New ABCG2 Transporter Inhibitors as Potential Anticancer Agents

**DOI:** 10.3390/ijms24010725

**Published:** 2022-12-31

**Authors:** Simone Di Micco, Veronica Di Sarno, Martina Rossi, Vincenzo Vestuto, Takumi Konno, Sara Novi, Mario Felice Tecce, Valeria Napolitano, Tania Ciaglia, Andrea Vitale, Isabel Maria Gomez-Monterrey, Giuseppe Bifulco, Alessia Bertamino, Carmine Ostacolo, Paolo Blasi, Alessio Fasano, Pietro Campiglia, Simona Musella

**Affiliations:** 1 European Biomedical Research Institute of Salerno, Via S. De Renzi 50, 84125 Salerno, Italy; 2 Department of Pharmacy, University of Salerno, Via G. Paolo II, 84084 Fisciano, Italy; 3 Department of Pharmacy and Biotechnology, Via San Donato 19/2, 40127 Bologna, Italy; 4 Center for Applied Biomedical Research (CRBA), University of Bologna, 40126 Bologna, Italy; 5 Department of Pediatrics, Mucosal Immunology and Biology Research Center, Massachusetts General Hospital, Boston, MA 02114, USA; 6 Department of Pediatrics, Division of Pediatric Gastroenterology and Nutrition, Massachusetts General Hospital for Children, Boston, MA 02114, USA; 7 Pineta Grande Hospital, Via Domiziana, km 30/00, 81030 Castel Volturno, Italy; 8 Department of Pharmacy, University of Naples Federico II, Via D. Montesano 49, 80131 Napoli, Italy

**Keywords:** drug discovery, in silico studies, multidrug resistance, multicellular tumor spheroids, ATP-binding cassette

## Abstract

Different molecular mechanisms contribute to the development of multidrug resistance in cancer, including increased drug efflux, enhanced cellular repair mechanisms and alterations of drug metabolism or drug targets. ABCG2 is a member of the ATP-binding cassette superfamily transporters that promotes drug efflux, inducing chemotherapeutic resistance in malignant cells. In this context, the development of selective ABCG2 inhibitors might be a suitable strategy to improve chemotherapy efficacy. Thus, through a multidisciplinary approach, we identified a new ABCG2 selective inhibitor (**8**), highlighting its ability to increase mitoxantrone cytotoxicity in both hepatocellular carcinoma (EC**_50_**from 8.67 ± 2.65 to 1.25 ± 0.80 μM) and transfected breast cancer cell lines (EC**_50_**from 9.92 ± 2.32 to 2.45 ± 1.40 μM). Moreover, mitoxantrone co-administration in both transfected and non-transfected HEK293 revealed that compound **8** notably lowered the mitoxantrone EC_50,_ demonstrating its efficacy along with the importance of the ABCG2 extrusion pump overexpression in MDR reversion. These results were corroborated by evaluating the effect of inhibitor **8** on mitoxantrone cell uptake in multicellular tumor spheroids and via proteomic experiments.

## 1. Introduction

Despite the continuous advancement in the treatment of cancers and the progressive introduction of new protocols, including surgery, radiation therapy, combination therapy and laser therapy, chemotherapy remains the most used option. Although cancer cells are initially susceptible to chemotherapy, they can develop resistance through different mechanisms over time [1]. Moreover, certain cancer cells are characterized by pre-existent or intrinsic resistance to chemotherapeutics [2]. This is the reason why chemoresistance is one of the leading causes of antitumor therapy failure [3]. The progress of genomic and proteomic techniques allowed to better understand the intracellular mechanisms involved in drug-resistant cells development, selection, and proliferation [4,5]. Multiple mechanisms could be involved in the emergence of intrinsic or acquired multidrug resistance (MDR), including drug inactivation, apoptosis inhibition, target alteration, stimulation of DNA repair mechanisms and enhanced drugs efflux, and metabolism of xenobiotics [4,5,6].

Among these, alterations affecting surface proteins involved in cellular transport are particularly important [7,8]. Increased expression of membrane transporters (ABC: ATP Binding Cassette) is responsible for sustained drug extracellular transport [9]. ABCs belong to a superfamily of proteins that are responsible for the translocation of their substrates across the cell membranes, including several small inorganic or organic molecules, in addition to metal ions, lipids, polypeptides and proteins. ABC transporters include 49 proteins, arranged in seven subfamilies (ABCA to ABCG). In humans, the expression of 3 ABC transporters [ABCB1 (P-glycoprotein 1), ABCC1 (Multidrug Resistance-associated Protein 1) and ABCG2 (Mitroxantrone Resistance Protein)] has been associated with drug-resistance [10,11]. Overexpression of the P-glycoprotein 1 coding gene has been associated with failures in the treatment of leukemia, and kidney, liver, prostate, lung, and breast cancers [12]. At the same time, the recurrence of different cancers, such as leukemia [13], and lung [14], pancreatic [15], ovarian [16], or breast cancer [17], was already connected to the overexpression of ABC transporters. Since its discovery in 1998 [18,19,20], ABCG2 protein was observed to play a leading role in this field, conferring resistance to a broad spectrum of chemically unrelated anticancer drugs, including camptothecins, mitoxantrone, and tyrosine kinase inhibitors (TKIs) [21,22].

In this scenario, the development of a selective ABCG2 inhibitor might be a suitable strategy to tackle MDR. The coadministration of ABCG2 transporter inhibitors with an anticancer drug has been evaluated as a relevant approach to overcome MDR and improve cancer treatment [6]. A relatively small number of potent ABCG2 inhibitors are currently available, and the development of more potent and selective compounds is still urgently needed [23,24,25].

**Figure 1:**
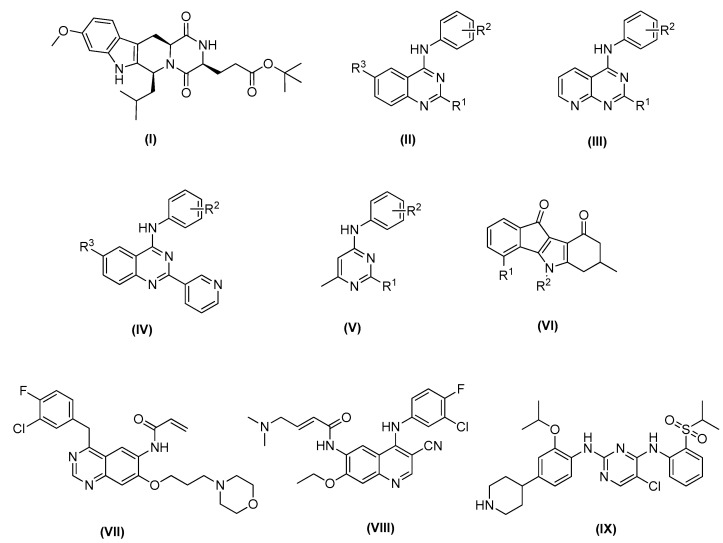
Recent ABCG2 inhibitors: Ko143 (**I**); small molecules based on 1,4,6-substituted quinazoline scaffold (**II**), 2,4-disubstituted pyridopyrimidine scaffold (**III**), 4-anilino-2-pyridyl quinazoline scaffold (**IV**) and 4-methyl-pyrimidine scaffold (**V**); general structure of indeno [1,2-b]indole-9,10-dione (**VI**); new multitarget tyrosine kinase inhibitors (TKIs) reversers of ABCB1-, ABCC1-, and ABCG2-mediated MDR, canertinib (**VII**), pelitinib (**VIII**) and ceritinib (**IX**).

Fumitremorgin C was the first ABCG2 inhibitor identified, and it was characterized by a low effective concentration (EC_50_ around 1−5 μM) as well as notable neurotoxicity [26,27]. Therefore, it failed clinical development [28]. In later studies, Ko143 (Figure 1 compound I), one of the fumitremorgin C metabolites, was identified as a potent and selective inhibitor of ABCG2, with an EC_50_ around 10 nM, although it was not stable in mouse plasma when orally administered [27].

Recently, small molecules based on 2,4,6-substituted quinazoline [29], 2,4-disubstituted pyridopyrimidine [30] and 4-anilino-2-pyridyl quinazolines and 4-anilino-2-pyrimidine [31] cores have been reported to be highly potent and nontoxic inhibitors of ABCG2 (Figure 1 compounds II–V). In addition, a series of indeno [1,2-b]indole-9,10-dione derivatives, synthesized as human casein kinase II (CK2) inhibitors (Figure 1 compound VI), have been reported for inhibiting mitoxantrone efflux by selectively blocking ABCG2 [32].

For ABCG2, cross-resistance against several structural different antineoplastic agents has been described, such as camptothecins, epipodophyllotoxins, mitoxantrone, or tyrosine kinase inhibitors (TKIs) (Figure 1, compounds VII–IX) [33]. Canertinib represents the very first TKI proven to interact with ABCG2 in an inhibitory way [17]. Then, other different tyrosine kinase inhibitors structurally related to canertinib, including pelitinib [14], and ceritinib [34], have become new multitarget reversers of ABCB1-, ABCC1-, and ABCG2-mediated MDR.

In the present work, we discuss the process leading to the discovery of new ABCG2 selective inhibitors that might be helpful to improve the efficacy of anticancer drugs, while decreasing their doses and thus side effects and the risk for MDR.

## 2. Results

### 2.1. In-Silico Analysis

**Figure 2:**
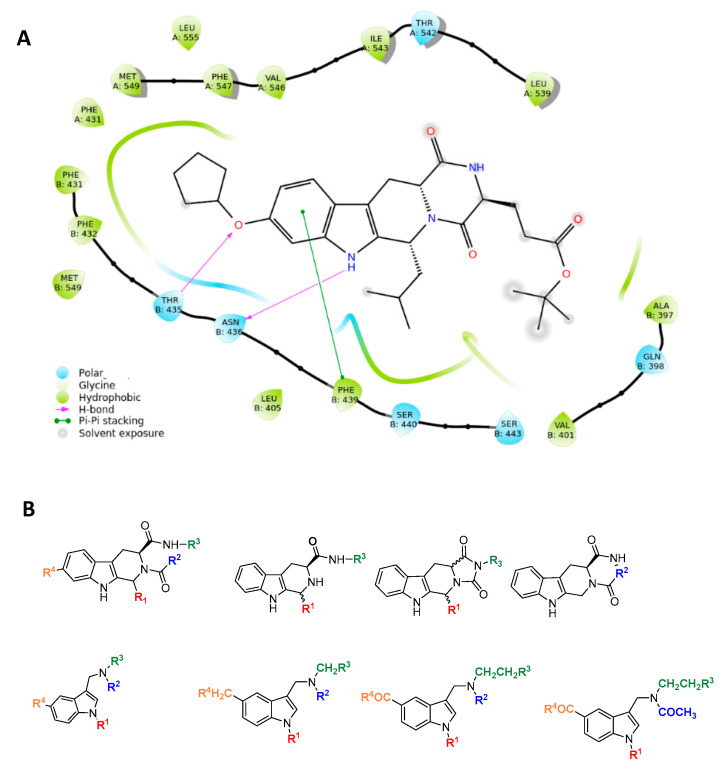
Rational design of ABCG2 inhibitors: (**A**) 2D interaction diagram of MZ29-ABCG2 complex (PDB ID: 6ETI); (**B**) tetrahydro-β-carboline and indole-based scaffolds.

The resolved structure of the complex between ABCG2 and the Ko143 derivative MZ29 [35] led us to design a focused library. Specifically, the experimental ligand-protein structure suggested three key intermolecular interactions via the tetracyclic portion of MZ29: two hydrogen bonds with side chains of T435 and N436 and a π-stacking with F439 by the indole moiety of the polycyclic core (Figure 2).

**Figure 3:**
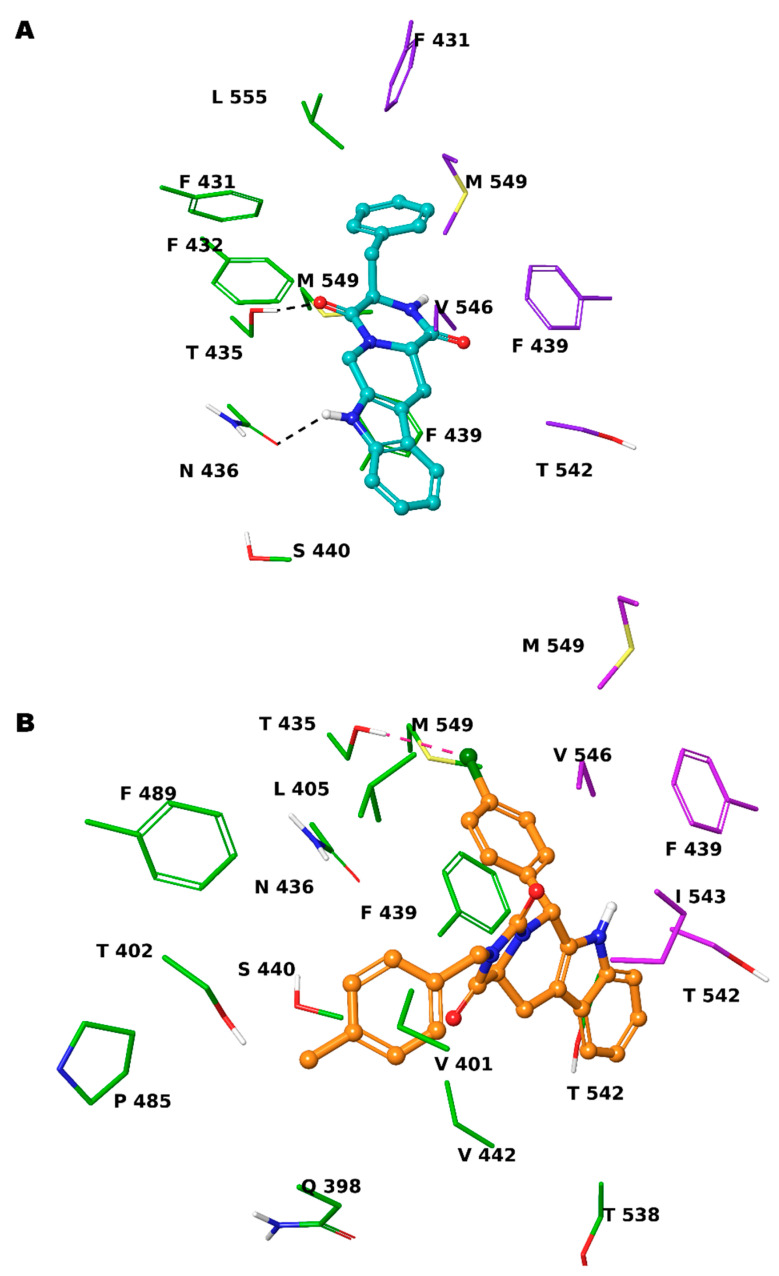
Three-dimensional model of the interactions given by **8** (**A**) and **13a** (**B**) with ABCG2. The protein is depicted by tube, with the following color-code: C, green (chain A) and violet (chain B); polar H, white; N, dark-blue; O, red; S, yellow). The small molecules are represented by sticks (teal for **8**, orange for **13a**) and balls (colored: C, as for the sticks; polar H, white; N, dark-blue; O, red; Cl, forest green). The dashed green and pink lines indicate the hydrogen and halogen bonds, respectively, between the ligand and protein.

Based on these observations, we specifically selected the tetrahydro-β-carboline [36] and indole-based scaffolds [37] from our in-house library to build a targeted molecule collection, in order to trace the main intermolecular interactions, which were experimentally identified. Specifically, we decorated both the molecular frameworks with different substituents (aliphatic/aromatic groups and H-bond donor/acceptors), considering their synthetic feasibility, and built a library of 6.261 molecules. As a protein model, we used two available experimental structures of ABCG2 (PDB IDs: 6ETI as Model A and 6FEQ as Model B), because structural experiments revealed different spatial arrangements of N436 upon ligand binding [38,39,40]. In silico screening was performed for the library built with this approach for both Models A and B, and the docked poses were filtered by considering the above-mentioned key intermolecular interactions and molecular diversity. From our analysis, 6 tetrahydro-β-carboline-based and 7 indole-based molecules were filtered and synthesized for experimental testing (Schemes S1–S3). For the sake of simplicity, we describe only the detailed interaction given by docked poses of **8** and **13a**, as both compounds showed the most promising biological profiles (see below). The docked pose of **8** showed proper accommodation into the binding site of ABCG2 (Figure 3A). The aromatic indole moiety establishes a staggered stacking with F439 and donates a hydrogen bond with its NH to side chain of N436 (Figure 3A). The carbonyl group at C-4 is H-bonded to the side chain of T435 (Figure 3A). It is noteworthy that these interactions are also observed for the co-crystallized MZ29 into the ABCG2 binding pocket. The benzyl group gives van der Waals contacts with A:F431, A:F432, A:M549, B:F431, B:M549, B:I550, and B:L555. The tetrahydropyridine also contributes to the van der Waals interactions with B:F439, B:T542, and B:V546. Interestingly, **13a** provides two halogen bonds with T435 instead of a hydrogen bond, while maintaining a parallel-displaced stacking with F439 (Figure 3B). A second halogen bond is established with A:M549 (Figure 3B). Unlike MZ49, the 4-methylbenzyl group of **13a** is accommodated into a hydrophobic cavity delimited by residues A:Q398, A:V401, A:L405, A:P485, and A:F489, exploring new binding cavity portions. Similarly, the indole moiety is involved in the van der Waals interactions A:V442, A:T538, A:T542, A:F439, B:F439, and B:T542 (Figure 3B), providing extra contacts than MZ29.

### 2.2. Biological Investigation

**Figure 4:**
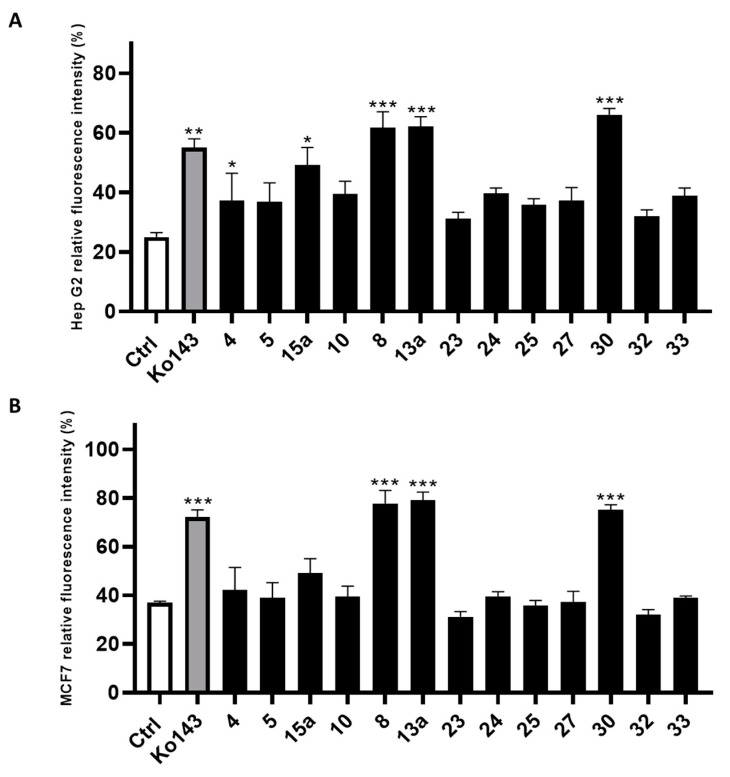
Inhibitory effect of screened compounds toward ABCG2 in cell lines Hep G2 (**A**) and MCF7 (**B**) in a Hoechst 33,342 assay at the concentration of 20 μM. Ko143 was used as positive control at the same concentration of the tested small molecules. For each compound, three independent experiments were performed, and the standard deviation are expressed as error bars. *, **, *** denote *p* < 0.05, *p* < 0.01 and *p* < 0.001, respectively, vs. the ctrl.

The compounds filtered through the virtual screening protocol were evaluated for their inhibitory activity towards ABCG2 transporters using Hep G2 and transfected MCF7 cells, overexpressing ABCG2 protein, by using the well-known ABCG2 inhibitor Ko143 as the reference compound [41,42]. We performed the Hoechst 33,342 microplate assay, slightly modifying the protocol described in the literature [29,43]. In detail, the dye Hoechst 33,342 accumulates into the cells, binding to the DNA minor groove, and then, the dye can be fluorometrically detected. As Hoechst 33342 is a substrate of ABCG2, it can be extruded from the cell and its fluorescence decays. Thus, the measured fluorescence in the presence of the designed small molecules allowed the determination of their inhibitory efficacy against ABCG2. The results from the Hoechst 33342 accumulation assay conducted on Hep G2 cells are reported in Figure 4A, while the results of the same assay performed on the MCF7 cell line are reported in Figure 4B. Compounds **8**, **13a** and **30** showed the best inhibition of ABCG2 on both the cell lines. It is noteworthy that **8**, **13a** and **30** had a slightly better inhibitory activity than the reference compound Ko143. The remaining compounds showed an ABCG2 inhibition profile lower than that of Ko143.

Considering the remarkable activity showed by compounds **8**, **13a** and **30** both in Hep G2 and MCF7 cells, we questioned whether this activity was eventually related to their inherent cytotoxicity. Accordingly, the cytotoxicity of the three molecules was evaluated in Hep G2, transfected MCF7 and non-tumorigenic MCF 10A cell lines using the MTT assay (Table 1). The toxicity of the compounds was determined by titrating the small molecules in the 1–100 µM concentration range and assessing cell viability 24 h after the administration using MTT assays. Then, 0.1% (*v*/*v*) and 10% (*v*/*v*) DMSO were used as the negative and positive controls, respectively. Compounds **8** and **13a** showed no cytotoxic activity, with EC_50s_ >100 µM in all the cell lines tested (Table 1). Conversely, compound **30** showed a non-negligible toxicity (28.21 ≤ EC_50_ ≤ 36.12 µM) in the cell lines tested (Table 1). These results prompted us to further investigate compounds **8** and **13a**. Thus, functional assays on these two compounds were performed, investigating their putative ability to increase the mitoxantrone cytotoxicity as result of ABCG2 inhibition in both Hep G2 and transfected MCF7 cell lines.

**Figure 5:**
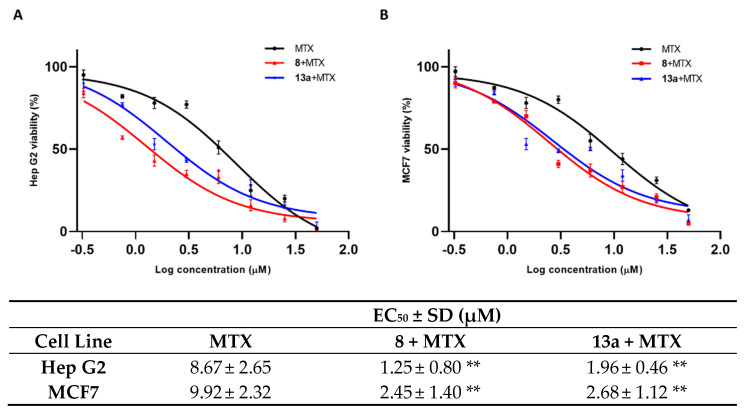
The EC_50_ of MTX alone and in co-administration with **8** (red peak) and **13a** (blue peak), at a concentration of 1 µM in Hep G2 (**A**) and MCF 7 (**B**) cell lines. Standard deviation is expressed as error bars. Mean EC_50_ values are reported in the corresponding table. ** denotes *p* < 0.01 vs. ctrl.

As shown in Figure 5, the mitoxantrone EC_50_ for both cell lines is remarkably decreased when the tumor cells are pre-treated with 1 μM concentration of compounds **8** and **13a**. Both tested small molecules present a comparable modulation of mitoxantrone activity.

**Figure 6:**
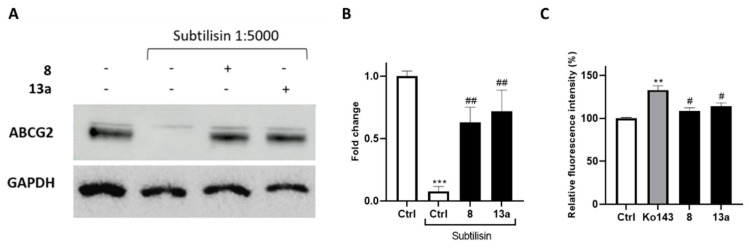
(**A**) lmmunoblotting analysis of the DARTS experiment revealing ABCG2 as the likely target of compounds **8** and **13a**, together with its densitometric analysis (**B**). GAPDH is resistant to subtilisin under these experimental conditions and was used as a loading control. (**C**) Selectivity assay of compounds exhibiting a reduction in the fluorescence signal compared to the non-selective reference Ko143 in the calcein-AM assay. For each compound, three independent experiments were performed, and the standard deviation is expressed as error bars. **, *** denote *p* < 0.01 and *p* < 0.001, respectively, vs. the ctrl; #, ## denote *p* < 0.05 and *p* < 0.01, respectively, vs. the positive ctrl/Ko143.

To further confirm the interaction of compounds **8** and **13a** with ABCG2, we performed drug affinity responsive target stability (DARTS) experiments. This compound-centered proteomic approach is based on limited proteolysis of cell lysates incubated with the small molecule of interest. This technique takes advantage of the interaction of a protein with a ligand, which dramatically increases protein stability thereby reducing its proteolytic susceptibility. The controlled proteolysis employs a low-specificity protease such as subtilisin. SDS-PAGE and subsequent Western blotting analysis (Figure 6A,B) revealed some degree of protection exerted by **8** and **13a** towards the ABCG2 protein. Next, compounds were further investigated in a calcein-AM (calcein-acetoxymethyl ester) microplate assay to determine their selectivity (Figure 6C) against the ATP-binding cassette family. Once administered, calcein-AM passively diffuses into the cells, and it is cleaved by the cytosolic esterases releasing the fluorescent calcein. It is known that the extrusion of calcein-AM is mediated by the ATP-binding cassette family, including ABCB1, but not ABCG2 [44,45]. Therefore, selective ABCG2 inhibitors should not cause fluorescence increase, as they are unable to inhibit the pumps responsible for the calcein extrusion. The Hep G2 cell line, which overexpresses the main ABC transporter, [46,47,48], was selected for these analyses. Ko143 was used as a reference compound considering its lack of selectivity at high concentrations [49,50].

The outcomes of calcein-AM assay revealed the selectivity vs. ABCG2 for **8** and **13a**, as not relevant increase in fluorescence was observed compared to the control.

**Figure 7:**
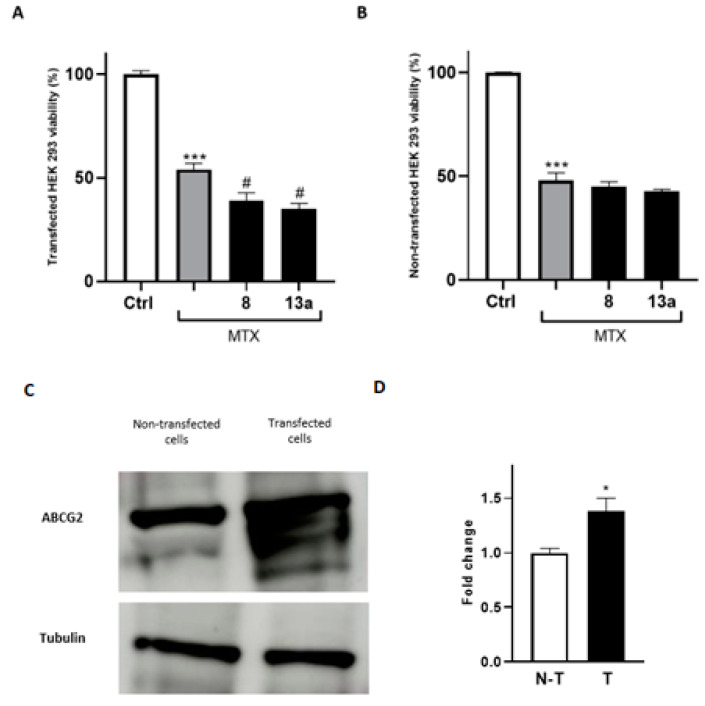
Cell viability assays using MTT conducted on the HEK293 wild type (**B**) and ABCG2-transfected cell lines (**A**). The EC_50_ of MTX was administered. For each compound, three independent experiments were performed, and the standard deviation is expressed as error bars. *, *** denote *p* < 0.05 and *p* < 0.001 vs. the ctrl, respectively; # denotes *p* < 0.05 vs. MTX. (**C**,**D**): Western blot analysis of HEK-293 cells and HEK-293 transfected cells with the ABCG2 plasmid. Tubulin was used as a loading control. N-T indicates non-transfected cells, while T indicates transfected cells.

To further prove that the antitumor efficacy of the compounds **8** and **13a** is mediated by their direct modulation of ABCG2, both transfected and non-transfected HEK293 cells were employed, as the questioned response is enhanced in systems overexpressing the protein of interest. Firstly, HEK293 cells were transfected with the gene expressing the receptor. Then, to confirm the effectiveness of transfection, we used a proteomic approach by performing a mass spectrometry analysis of anti-ABCG2-reacting bands resolved via gel electrophoresis. As expected, lysates of transfected HEK293 cells with the ABCG2 receptor showed immune-reacted bands that were excited from a paralleled-run gel electrophoresis and submitted to mass spectrometry analysis. These results confirmed the overexpression of ABCG2 gene and, therefore, were suitable to test the ability of **8** and **13a** to improve mitoxantrone potency in co-administration experiments. As shown in Figure 7, the mitoxantrone cytotoxicity significantly increased when co-administered with **8** and **13a**, compared to the administration of the well-known antitumor agent alone. Moreover, the enhancement of the mitoxantrone cytotoxicity when co-administered with **8** and **13a** was significantly more pronounced in the transfected cell line than in the wild-type system, demonstrating the potential of the compounds in MDR reversion considering overexpressed extrusion pumps.

### 2.3. HepG2 Spheroid Production and Mitoxantrone Treatment

**Figure 8:**
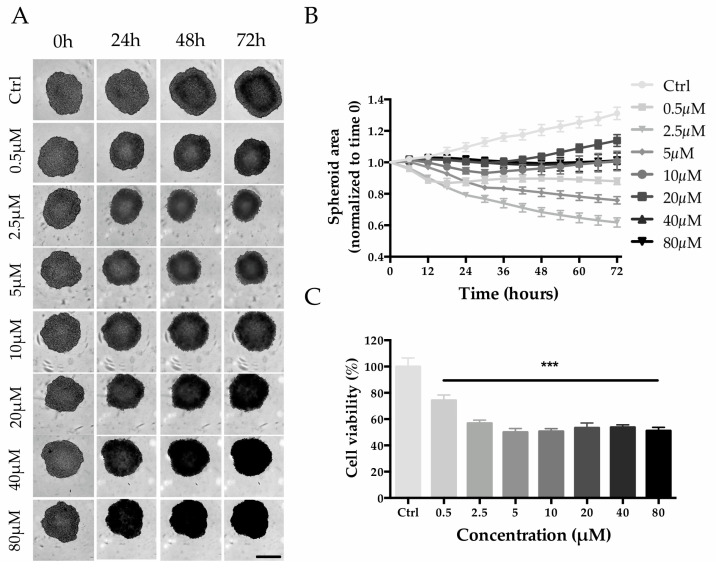
Effect of MTX on HepG2 spheroid size and cell viability. (**A**) Phase-contrast images (Incucyte^®^) of MTX-treated spheroids recorded every 24 h. Scale bar 400 µm. (**B**) Spheroid size calculated by using the Incucyte^®^ Zoom Software every 6 h. (**C**) Spheroid viability after 72 h of treatment with MTX at a final concentration of 80, 40, 20, 10, 5, 2.5, and 0.5 µM. Viability is expressed as the percentage of resazurin reduction with respect to the control. *** denotes *p* < 0.001 vs. the ctrl.

HepG2 cell seeding densities were screened to establish the optimal conditions for the generation of Multicellular Tumor Spheroids (MCTS). A seeding density of 2000 cells/well was selected for the experiment since it allowed the production of tight spheroids with a size of ~500 µm within 3 days of culture (Figure S1). Untreated spheroids (control group) increased in size by ~30% from day 3 to day 6, while when treated with 0.5 and 2.5 µM of MTX, the size decreased in a concentration-dependent manner (Figure 8A,B). Interestingly, even though MTX-treated spheroids were always smaller than that of the control group, higher MTX concentrations (from 5 to 80 µM) led to a minor reduction in size (Figure 8A,B). Despite that, a dose-response trend is appreciable with viability estimation: the viability decreased in a concentration-dependent manner up to 5 µM (0.5 µM, 74.3 ± 4.1%; 2.5 µM, 56.9 ± 2.4%; 5 µM, 50.1 ± 2.8%) and then remained at approximately 50% for higher concentrations (Figure 8C).

### 2.4. Effect of the Inhibitor (8) on MTX Uptake in HepG2 Spheroids

**Figure 9:**
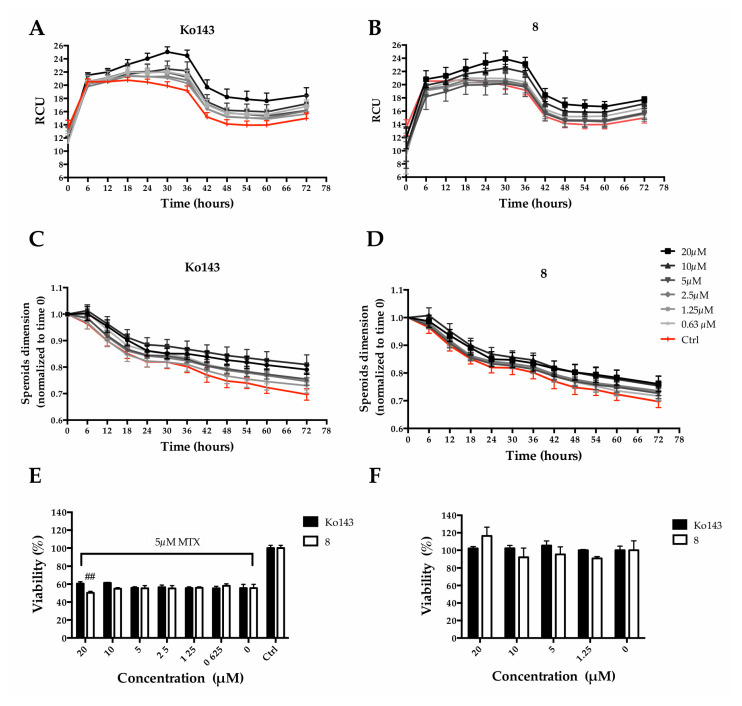
Effect of **8** or Ko143 on MTX uptake in Hep G2 spheroids. Spheroids were pre-treated (2 h) with Ko143 or alternatively with **8** at different concentrations (i.e., 20, 10, 5, 2.5, 1.25 and 0.625 µM) and then treated with MTX at a final concentration of 5 µM. Spheroid size was calculated through Incucyte^®^ Zoom Software (v2021) after pre-treatment with Ko143 (**A**) or **8** (**B**). Fluorescence intensity time course acquired with Incucyte^®^ for 72 h after mitoxantrone treatment with Ko143 (**C**) or **8** (**D**). Spheroid viability 72 h after treatment with (**E**) and without (**F**) MTX expressed as a percentage of resazurin reduction compared to the ctrl. ## denotes *p* < 0.01 vs. MTX alone.

We decided to mainly study the effects exerted by compound **8**. Particularly, we quantified MTX uptake, spheroid size and cell viability overtime. Ko143 was used as a comparison. MTX fluorescence was employed to quantify its accumulation within MCTS, while metabolic activity was used to estimate viability. An increase in MCTS fluorescence intensity, due to a higher MTX uptake, could be observed as a consequence of the pretreatment with the Ko143 or **8** (Figure 9A,B), indicating a partial block of the protein-mediated efflux due to the inhibitors. Fluorescence accumulation was related to the inhibitor concentrations: a higher inhibitor concentration produced greater fluorescence. At the same time, the pre-treatment caused a slight increase in MCTS size in a dose-dependent manner (Figure 9C,D). Although counterintuitive, the increase in size is reasonably due to a reduction in the spheroid stiffness as a consequence of a higher MTX uptake. However, a significant reduction of spheroid viability (9.46 ± 2.9% of viability reduction) could be observed when pre-treating with **8** at 20 µM, the highest tested concentration (Figure 9E). It is worth noting that Ko143, the well-known ABCG2 inhibitor used as reference, at the same concentration, did not show the same effect on viability.

To rule out a possible cytotoxic effect of **8**, cell viability assays were performed in the absence of mitoxantrone. Cell viability was not affected either with Ko143 treatments or with **8** at concentrations up to 20 µM (Figure 9F).

## 3. Discussion

In the context of drug resistance in cancer cells, ABCG2 has the feature of extruding a wide spectrum of chemically unrelated chemotherapeutic drugs, such as mitoxantrone, camptothecins, and tyrosine kinase inhibitors (TKIs). Thus, the coadministration of a selective ABCG2 inhibitor can be a proper approach to overcome MDR and improve chemotherapy. As a limited number of potent ABCG2 inhibitors are currently available, the identification of more potent and selective compounds is still urgently required.

The inspection of ligand-protein interactions provided by the experimentally resolved structure of ABCG2 bound to Ko143 analogue MZ29 inspired the design of a focused library. From the polycyclic structure of MZ29, the indole moiety establishes primary intermolecular interactions for the complex line-up. These structural observations guided the selection of suitable molecular frameworks from our in-house compound collection to build a focused library: the tetrahydro-β-carboline scaffold, preserving the polycyclic structure as MZ29, and the indole-based scaffold as a simplified structural requirement to target ABCG2. From virtual screening, 13 molecules, endowed with the tetrahydro-β-carboline and indole-based scaffolds, were filtered for biological investigation. Experimental tests showed that all the tested compounds presented an inhibitory activity, but three small molecules (**8**, **13a** and **30**) gave a slightly superior activity than Ko143. The small molecule **8** conserved the same tetracyclic moiety of MZ29 but differed considering the inversion of chirality of C-3 and the absence of a substituent at C-12. These structural features of **8** induced a docked pose that was rotated by 180° with respect to the conformation of MZ29, while preserving the crucial H-bonds with T435 and N436. Moreover, a deeper accommodation and tighter contacts are observed for **8** considering MZ29, including the hydrogen bonds with T435 and N436. Indeed, a reduced distance is found between interacting moieties of **8** and the side chains of T435 and N436: 1.78 and 2.17 Å, respectively, against 2.43 and 2.40 Å of MZ29. The reduction of the diketopiperazine to a five-membered ring and the inversion of C-12 inserting the p-Cl-phenyl in **13a** were not detrimental to the activity. Indeed, two halogen bonds are observed along with a favorable π-stacking, while the remaining structural portions gave wide van der Waals contacts with macromolecular counterparts, and also explore new binding site spaces not observable by the reference compound. Unlike the polycyclic core of MZ29, compound **30** featured an indole-based scaffold. The experimental outcomes demonstrated that this simplified cyclic molecular system is suitable for designing a new ABCG2 inhibitor and can be also used as a molecular seed for developing multitargeting compounds. However, **30** showed an inherent cytotoxicity against Hep G2 and MCF7 unlike **8** and **13a**. Thus, compounds **8** and **13a** were chosen for further investigation. Interestingly, both compounds notably lowered the EC_50_ of mitoxantrone in both Hep G2 and MCF7 cell lines. As **8** and **13a** lacked cytotoxicity against the considered cell lines, we ascribed the improvement of the antitumoral profile of mitoxantrone to their inhibitory activity against ABCG2. These data are well integrated by the investigation of selectivity vs. ABCG2 over other ATP-binding cassette by using DARTS and calcein-AM assays. Indeed, both independent assays proved the preference of binding towards ABCG2. With the aim of confirming the inhibitory effect of **8**, the size and cell viability of spheroids, along with MTX uptake, were evaluated overtime by using Ko143 as reference. Interestingly, an increase in spheroid size was observed and this effect can be ascribed to a reduction in the stiffness when MTX accumulated in the cells. Upon treatments with **8** and Ko143, an increase in MCTS fluorescence intensity was observed, due to a partial block of the pump efflux by these inhibitors with a consequent higher MTX uptake. It is noteworthy that a significant reduction in spheroid viability could be observed by treatment with **8** at 20 µM, whereas no effect was observed with Ko143 at the same concentration. Moreover, **8** did not have a cytotoxic effect on spheroid viability without MTX co-administration, further proving that its modulation of spheroid viability includes ABCG2 impairment and MTX accumulation.

Collectively, the reported data showed the identification of new lead compounds for developing selective and safer ABCG2 inhibitor to tackle drug resistance and improve current chemotherapy efficacy.

## 4. Materials and Methods

### 4.1. Computational Details

The three-dimensional structures of library compounds were sketched by using Build Panel of Maestro (version 11, Schrödinger, LLC., New York, NY, USA), and then, the small molecules were optimized through OPLS3 force field [51], Polak-Ribière conjugate gradient algorithm (maximum derivative <0.001 kcal/mol), and GB/SA (generalized Born/surface area) [52] as the solvent treatment of H_2_O. The whole library was processed by using LigPrep [53], with the ionizer option and accounting for the protonation states at pH of 7.0 ± 1.0. Two electron microscopy structures of ABCG2 (PDB IDs: 6ETI as Model A; 6FEQ as Model B) were processed using the Protein Preparation Wizard [54,55]: hydrogen addition; bond order assignment; checking for a missing side chain and loop; checking of alternate positions of the residues; assignment of side chain charge (pH 7.0 ± 1.0); and H-bond network improvement by using the optimize option. The H_2_O molecules were removed. Molecular docking predictions were carried out by using Glide (v. 7.2, Schrödinger, LLC., New York, NY, USA) [56,57,58]. The docking protocol was validated by redocking the co-crystallized MZ29 with ABCG2 and overlapping the docked and experimental poses (Figure S2; RMSD = 0.574 Å) [59,60,61]. The inner and outer grid boxes were sized 10 Å and 16 Å, respectively, with center coordinates: −6.38 (x), −8.11 (y), −0.08 (z). Firstly, we used Standard Precision (SP), applying default parameters with the enhanced sampling option for conformer generation and expanded sampling for the selection of the initial poses. One pose per ligand was generated and employed as the input conformations for the Extra Precision (XP) Glide mode run of predictions with both Models A and B, considering the halogen atoms to be the acceptor and donor of bonds. The ligands were treated as flexible, allowing only the trans conformation for the amide bond, and the sampling of nitrogen inversion and ring conformations (energy cut-off = 2.5 kcal/mol). The enhanced sampling option was utilized, keeping 10,000 poses/ligand for the initial step of docking and taking 1000 poses per ligand for energy minimization. For each small molecule, 1000 maximum output conformations were maintained by applying 0.15 as the partial charge cut-off and 0.8 as the scaling factor for the van der Waals radii. Post-docking optimization was executed on docked conformations, considering 10 as the maximum number of poses and utilizing 0.5 kcal/mol as the cut-off to filter for the obtained minimized poses. The following energy contributions were accounted for: aromatic-H and halogen bonds (as donor and acceptor); the reward of intramolecular H-bonds; and Epik state penalty. Maestro (version 11, Schrödinger, LLC., New York, NY, USA) was employed for the molecular modelling study and for figure production.

### 4.2. Chemistry

General: All reagents and solvents used were purchased from Sigma-Aldrich (Milan, Italy) unless otherwise stated. Reactions were performed under magnetic stirring in round-bottomed flasks unless otherwise noted. Moisture-sensitive reactions were conducted in oven-dried glassware under nitrogen stream, using freshly distilled solvents. TLC analysis of reaction mixtures was performed on precoated glass silica gel plates (F254, 0.25 mm, VWR International), while crude products were purified by with the Isolera Spektra One automated flash chromatography system (Biotage, Uppsala, Sweden), using commercial silica gel cartridges (SNAP KP-Sil, Biotage). NMR spectra were recorded on a Bruker Avance 400 MHz apparatus, at room temperature. Chemical shifts were reported in δ values (ppm) relative to internal Me_4_Si for ^1^H and ^13^C NMR. J values were reported in hertz (Hz). ^1^H NMR peaks were described using the following abbreviations: s (singlet), d (doublet), t (triplet), and m (multiplet). HR-MS spectra were recorded using an LTQ-Orbitrap-XL-ETD mass spectrometer (Thermo Scientific, Bremen, Germany), equipped with an ESI source. All the final compounds showed a purity of ≥95% as assessed via RP-UHPLC-PDA analysis, performed using a Nexera UHPLC system (Shimadzu, Kyoto, Japan) consisting of a CBM-40 lite controller, two LC-40B X3 pumps, an SPD-M 40 photo diode array detector, a CTO-30A column oven, and a SIL-40C X3 autosampler. The chromatographic analysis was accomplished on a Kinetex^®^ Evo C18 column, 150 × 2.1 mm × 2.6 µm (Phenomenex^®^, Bologna, Italy) maintained at 40 °C. The optimal mobile phase consisted of 0.1% HCOOH/H_2_O *v*/*v* (A) and 0.1% HCOOHIN *v*/*v* (B) delivered at a constant flow rate of 0.3 mL/min -1. Analysis was performed in gradient elution as follows: 0–20.00 min, 5–95% B; 20.00–25.00 min, isocratic to 95% B; then 5 min for column re-equilibration. Data acquisition was set in the range of 190–800 nm and chromatograms were monitored at 254 nm.

### 4.3. 2D Cell Cultures and Transfection

The human hepatoma Hep G2 cell line was obtained from GMP-IST cell bank (Genova, Italy) and was grown in Eagle’s Minimum Essential Medium (EMEM) supplemented with 10% (*v*/*v*) fetal bovine serum, 2 mM L-glutamine, 1% (*v*/*v*) non-essential amino acids, 100 U/mL penicillin, and 0.1 mg/mL streptomycin.

The human breast cancer MCF 7 and non-tumorigenic epithelial MCF 10A cell lines were purchased from the American Type Culture Collection (ATCC; Manassas, VA, USA). MCF 7 cells were cultured in Dulbecco’s Modified Eagle Medium (DMEM, 4500 mg/mL glucose) supplemented with 10% (*v*/*v*) fetal bovine serum, 2 mM L-glutamine, 100 U/mL penicillin, and 0.1 mg/mL streptomycin.

MCF7 transient transfection (ABCG2, NM_004827, Human Tagged ORF Clone, Origene) was performed with TransIT-LT1 Transfection Reagent (Mirus) according to the manufacturer’s protocol. Transfected cells were used after 48 h.

MCF 10A cells were maintained in 1:1 mixture of DMEM and Ham’s F12 medium supplemented with 10% (*v*/*v*) fetal bovine serum, 2 mM L-glutamine, human recombinant epidermal growth factor (20 ng/mL), insulin (10 mg/mL), cholera toxin (100 ng/mL) and hydrocortisone (5 mg/mL).

The human embryonic kidney HEK293 cell line was purchased from the American Type Culture Collection (ATCC; Manassas, VA, USA). HEK293 cells were cultured in Dulbecco’s Modified Eagle Medium (DMEM, 4500 mg/mL glucose) supplemented with 10% (*v*/*v*) fetal bovine serum, 2 mM L-glutamine, 100 U/mL penicillin, and 0.1 mg/mL streptomycin. HEK293 transient transfection was performed as described above.

Cells were routinely grown in culture dishes (Corning, Corning, New York, NY, USA) in an environment containing 5% CO_2_ at 37 °C and passaged at confluence using a solution of 0.025% trypsin and 0.01% EDTA. In each experiment, cells were placed in a fresh medium, cultured in the presence of synthesized compounds, and followed for further analyses. All experiments were performed in triplicate.

### 4.4. 2D Cell Viability Assay

Cell viability was evaluated by measuring mitochondrial metabolic activity using a colorimetric assay based on the reduction of 3-[4,5-dimethylthiazol-2,5-diphenyl-2H-tetrazolium bromide (MTT) to purple formazan. Briefly, Hep G2 (8 × 10^3^ cells/well), MCF7 and MCF 10A (5 × 10^3^ cells/well) were plated into 96-well plates containing 100 μL of the medium; after 24 h of growth to allow attachment to the wells, compounds were added at various concentrations (from 0.1 to 100 μM) for 24 h. Then, cells were replaced with a fresh medium containing 0.5 mg/mL MTT. Cells were incubated at 37 °C for 4 h. After that, 100 μL per well of 0.1 M isopropanol/HCl solution was added to dissolve the formazan crystals. The absorbance was measured at 570 nm using a microplate reader (Multiskan Go, Thermo Scientific, Waltham, MA, USA). Cell viability was expressed as a percentage relative to the untreated cells cultured in medium with 0.1% DMSO and set to 100%. The EC_50_ values were calculated using GraphPad Prism 8.0 software by nonlinear regression of the dose-response inhibition.

### 4.5. Hoechst 33,342 Accumulation Assay

Hep G2 (2 × 10^4^ cells/well) and transfected MCF7 (8 × 10^3^ cells/well) cells were seeded into 96-well plates and allowed to attach overnight to the surface of the black 96-well ViewPlate (PerkinElmer, Waltham, Massachusetts, USA). After 24 h, the test compounds (20 μM) were administered for 2 h to allow the blocking of the ABCG2 transporter. Then, the culture medium was removed, and the cells were incubated with a loading suspension (DMEM without phenol red, supplemented with Hoechst 33,342 at a final concentration of 3 μM) for 30 min. The vehicle DMSO (0.1%) served as a negative control. Afterwards, the cells were washed with PBS twice to remove residual dye. The relative fluorescence intensities (λ_exc_ 340 nm, λ_em_ 460 nm) were determined after 1 h using a PerkinElmer EnSight multimode plate reader. The data were compared to the fluorescence intensity in the absence of an ABCG2 inhibitor (negative control) and the response elicited by the Ko143 (positive control). Errors were expressed as the standard deviation (SD).

### 4.6. Mitoxantrone and Inhibitor Treatment on 2D Cell Cultures

Hep G2 (8 × 10^3^ cells/well) and transfected MCF7 (8 × 10^3^ cells/well) cells were grown in 96-well plates and pre-treated with test inhibitors for 2 h, and then, the EC_50_ of mitoxantrone was administered for 24 h. The EC_50_ values were calculated using GraphPad Prism 8.0 software via nonlinear regression of dose-response inhibition.

### 4.7. Drug Affinity Responsive Target Stability (DARTS) and Target Identification

The validation of the target of **8** and 1**3a** was performed by performing drug affinity responsive target stability (DARTS) experiments. To identify the target protein Hep G2, living cells were first plated, and after their adhesion, they were incubated with compounds at concentration corresponding to the respective EC_50_ for 2 h. After the treatments, cells were collected and total proteins were extracted by using the lysis buffer (20 mM Tris-HCl pH 7.5, 150 mM NaCl, 1 mM Na_2_EDTA, 1 mM EGTA, 2% NP-40, 1% sodium deoxycholate, 1x protease, and phosphatase inhibitor cocktail) for 30 min. The protein concentration was determined via the Bradford protein assay, using bovine serum albumin as the standard. Identical amounts of proteins (50 µg) were subjected to a limited digestion with subtilisin (1:5000 *w*/*w*). To stop the digestion, the resulting partially hydrolyzed protein mixtures were boiled in SDS-PAGE sample buffer (60 mM Tris-HCl pH 6.8, 2% SDS, 0.001% bromophenol blue, 10% glycerol, 2% 2-mercaptoethanol), and separated using 10% SDS-PAGE. Then, Western blotting analyses were conducted using an anti-ABCG2 antibody (1:1000, Abclonal, Woburn, Massachusetts, USA). GAPDH (1:1000, Santa Cruz Biotechnology) was used as the loading control. The signal was detected using an enhanced chemiluminescent substrate and LAS 4000 (GE Healthcare, Waukesha, WI, USA) digital imaging system.

### 4.8. Selectivity Assay

Selectivity over ABCG2 was determined by performing a calcein-AM assay to obtain information about the inhibitory activity of compounds towards ABCB1 and ABCC1 [62]. For this purpose, the assay was carried out on Hep G2 cell lines that overexpress different ABC proteins. Cells were seeded into 96-well plates (1 × 10^4^ cells/well), pre-treated with test inhibitors for 2 h (final concentration of compounds, 50 µM), and then washed and incubated with a calcein-AM solution (final concentration, 5 µM) for 30 min. Ko143 was used as a non-selective reference compound. The fluorescence signals (excitation/emission, 485 nm/520 nm) were read using a PerkinElmer EnSight multimode plate reader. The experiments were performed in triplicate. The data were reported as the mean ± SD using GraphPad Prism 8.0 software.

### 4.9. Spheroids Generation

Multicellular tumor spheroids (MCTS) from Hep G2 cell lines were generated by growing the cell suspensions in an ultra-low attachment 96-well plate (BIOFLOAT, faCellitate, Mannheim, Germany). Different cell-seeding densities (i.e., 8000, 4000, 2000, 1000, 500, and 250 cells per well) were compared to individuate the appropriate MCTS growth rate and size. To accelerate cell sedimentation and aggregation, centrifugation (300*g* × 1 min) was carried out immediately after seeding. Cells were then incubated at 37 °C, 5% CO_2_ for 3 days before treatment. A cell seeding density of 2000 cells per well was chosen for the following experiment.

### 4.10. Mitoxantrone and Inhibitor Treatment

MCTS (2000 cells/well) were grown for 3 days and then treated with different mitoxantrone solutions (i.e., 80, 40, 20, 10, 5, 2.5, and 0.5 µM) to individuate the most suitable concentration for inhibition experiments. MCTS were pre-treated for 2 h with Ko143 or **8** at different concentrations (i.e., 20, 10, 5, 2.5 1.25, and 0.625 µM) and then treated with 0.5 µM of mitoxantrone. DMSO was used as a control vehicle. Phase contrast and red fluorescence images were acquired at 3-h intervals for 3 days through a live-cells analysis system Incuyte^®^ (Sartorius, Goettingen, Germany). MCTS dimension and fluorescence intensity were analyzed through the Incucyte^®^ ZOOM software (v2021) [63].

### 4.11. 3D Cell Viability Assay

Cell viability was estimated using the metabolic indicator resazurin (TCI EUROPE, Zwijndrecht Belgium). A solution of resazurin at the final concentration of 200 µM was added 72 h after treatment and left to react for 16 h (37 °C, 5% CO_2_)_._ Then, 100 µL of the medium was collected for fluorescence acquisition (λ_exc_ 560 nm, λ_em_ 590 nm) using a plate reader (EnSpire^®^ Multimode Plate Reader, Perkin-Elmer, Waltham, MA, USA). Viability was reported as the percentage of resazurin reduction with respect to DMSO or mitoxantrone.

### 4.12. HEK293 Transfection and Mass Cell Analysis for Gene Expression Confirmation

The cell lysate sample and cell lysate transfected with ABCG2 were analyzed via Western blot analysis. Each 5 µL of the cell lysate samples was mixed with a sample buffer containing beta-mercaptoethanol and sodium dodecyl sulfate (SDS). Samples were then denatured at 99 °C for 5 min, and loaded into 4–20% tris-glycine gels with LI-COR Chameleon Duo ladder. After the gels electrophoresis was done, the gels were transferred onto PVDF membranes and rinsed with dH2O. The PVDF membranes were then blocked with 5% non-fat milk in Tris buffered saline (TBS), and incubated overnight at 4 °C with primary antibody diluted (1:1000) in TBS-Tween 0.1% (TBST). The membranes were then washed with TBST three times and incubated for 1 h at room temperature with a secondary antibody (1:20,000) that was diluted in TBST + 0.02% SDS. The Western blot signal was visualized in the 680 and 800 channels by using the Bio-Rad ChemiDoc MP Imaging system (Bio-Rad, Hercules, CA, USA). In this Western blot analysis, we used “RABBIT A5661 anti-ABCG2 antibodies as primary antibodies. For secondary antibodies, we used IRDye 800CW Donkey anti-Rabbit IgG Secondary Antibody (LICOR Biosciences, Lincoln, NE, USA). For mass spectrometry analysis for cell lysate samples, Western blot procedure was as previously described except for where noted here. After the gel electrophoresis was done, the gel was stained with Coomassie Blue (#24594, Thermo Fisher Scientific, Waltham, MA, USA). The gel bands around 100 kDa and 160 kDa were identified by using RABBIT A5661 and were excised and sent to the Taplin Mass Spectrometry Facility at Harvard Medical School. This analysis was performed according to the method previously reported [64]. We received the mass spectrometry analysis results from this facility.

### 4.13. Statistical Analysis

Data are reported as mean ± SD of results from three independent experiments. Statistical analysis was performed using an analysis of variance test, and multiple comparisons were made with the Bonferroni’s test with GraphPad Prism 8.0 software (San Diego, CA, USA). Significance was assumed at *p* < 0.05.

## Figures and Tables

**Table 1:** Overview of the EC_50_ results of the in vitro toxicity screening for **8**, **13a** and **30**. For each compound, at least three independent experiments were performed, and the results are given as EC_50_ ± SD.

	EC_50_ ± SD (µM)		
Compound	Hep G2	MCF7	MCF 10A
**8**	>100	>100	>100
**13a**	>100	>100	>100
**30**	28.21 ± 3.17	33.25 ± 2.63	36.12 ± 5.46

## Data Availability

The data presented in this study are available upon request to the corresponding author.

## Supplementary Materials

The following supporting information can be downloaded at: https://www.mdpi.com/article/10.3390/ijms24010725/s1. References [36,37,65] are cited in Supplementary Material.
