# Supplementary material for: In Silico Identification and In Vitro Evaluation of New ABCG2 Transporter Inhibitors as Potential Anticancer Agents
Source: Int J Mol Sci. 2022 Dec 31;24(1):725. doi: 10.3390/ijms24010725 (PMC9820944; doi:10.3390/ijms24010725)
Supplement: Supplementary file 1 [file ijms-24-00725-s001.zip › ijms-2103728-supplementary.pdf]

In silico identification and in vitro evaluation of new ABCG2 transporter inhibitors as potential anticancer agents

Table of content:

Page S1 – **Figure S1**: Phase contrast images of HepG2 spheroids
Page S2 – **Figure S2**: Superimposition of co-crystallized and docked poses of MZ29
Page S3-S13 – **Chemistry:** General methods and compounds characterization
Page S14-S*26* – **Figures S3-S15**: Analytical HPLC traces of compounds
Page S27– **References**

Supporting Information file

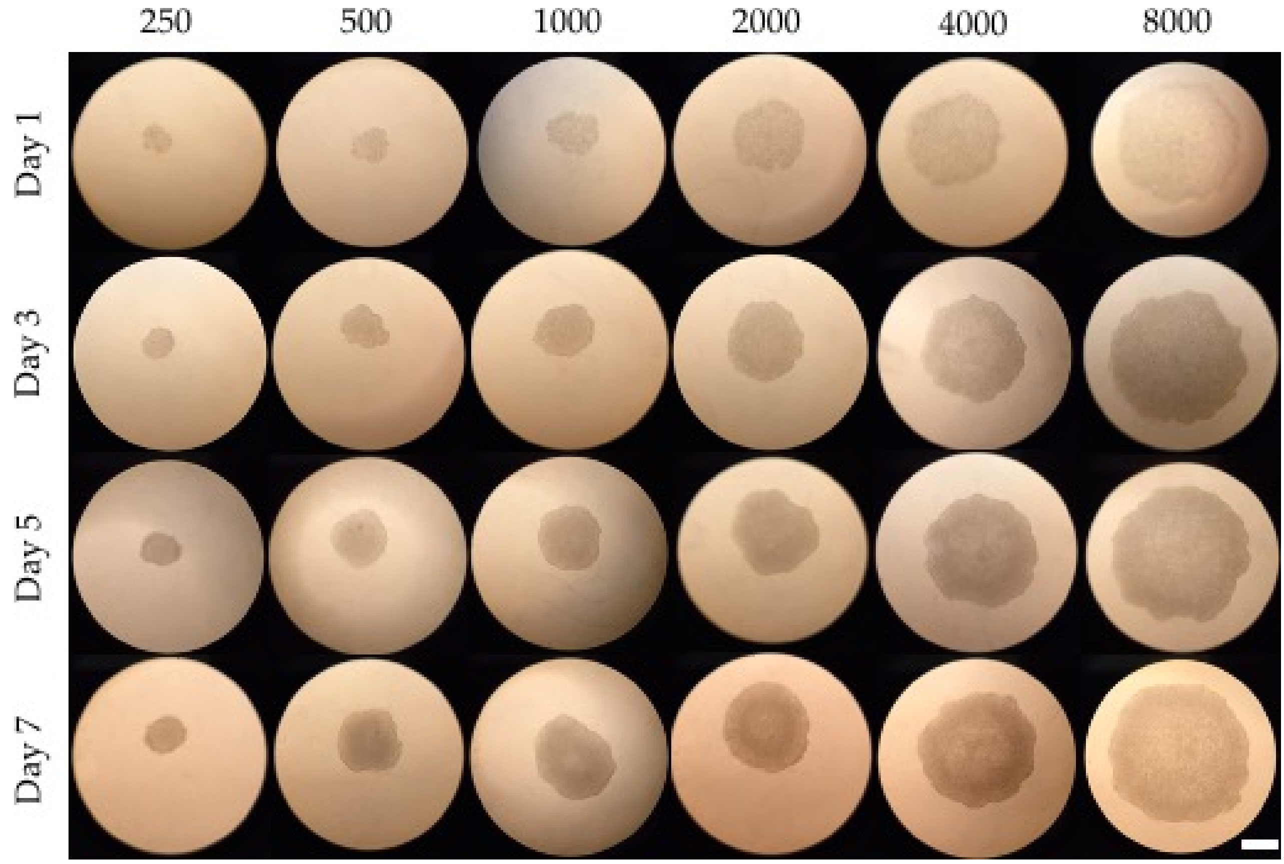

Figure S1. Sample phase contrast images of HepG2 spheroids acquired at different time points. MCTS were generated by seeding 250, 500, 1000, 2000, 4000, 8000 cells per well in Ultra Low attachment plates. Scale bar 400 μM.

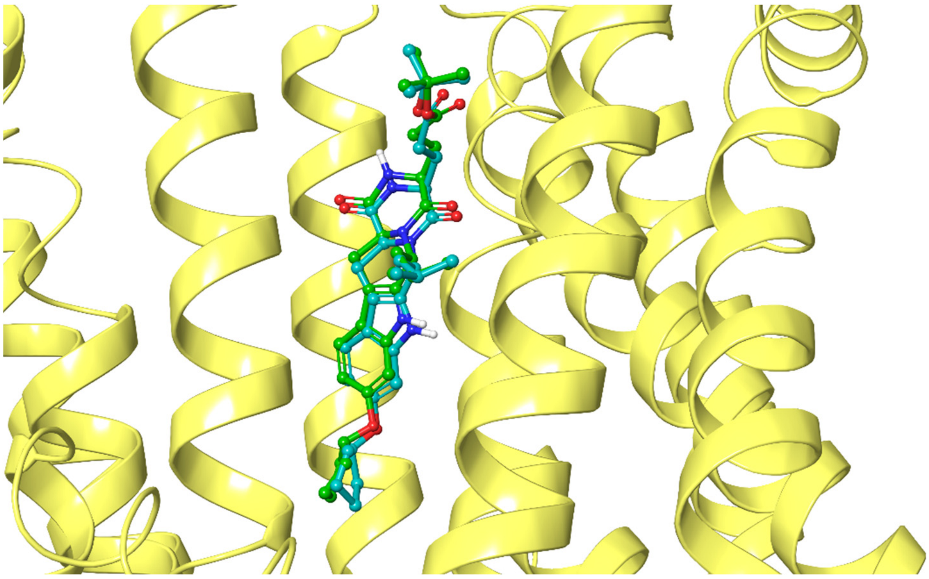

Figure S2. Superimposition (0.574 Å) of co-crystallized (green carbon, PDB ID: 6ETI) and docked pose (azure carbon) structures of MZ29. The protein is depicted by gold ribbons. The ligand is represented by sticks and balls (colored: C, as for the sticks; polar H, white; N, dark-blue; O, red).

1 Chemistry

1.1 Synthesis of THBC derivatives.

Tetrahydrobetacarbolines (THBCs) derivatives **4**, **5**, **8**, **10**, **13a** and **15a** were synthesized as depicted in Scheme 1. Starting from the reaction of NBoc-LTrp-OH with 4-F-benzylamine, using Pybop as coupling agents and DIPEA as base in a mixture of DCM/ DMF, gave the amide intermediate **2**, which was then deprotected in DCM/TFA (3:1 v:v) (**3**). The free amine **3** was subjected to a Pictet–Spengler reaction with isovaleraldehyde or methyl-4-oxobutanoate in the above-described conditions, leading to the formation of final THBC **4** and **5** (33–43% yields). On the basis of 2D NMR correlations and considering the fixed configuration at C-3 as S, we assigned the configurations to the THBC *cis* as **4**, **5** (1S,3S), respectively.

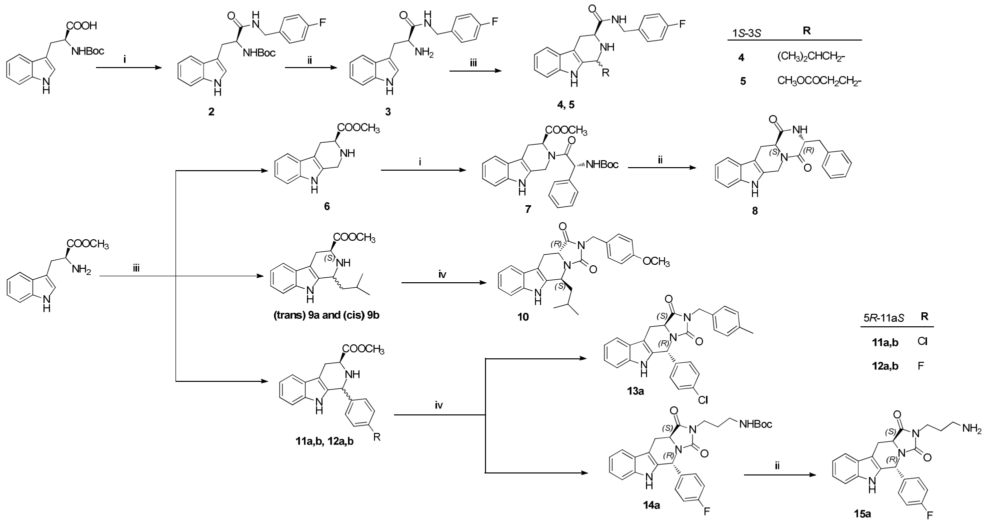

Scheme S1. Synthesis of THBC derivatives. i: 4-F-benzylamine (1.2 eq.) or N-Boc-D-Phe-OH (1.0 eq.), Pybop (1.2 eq.), DIPEA (2.4 eq.), DCM/DMF (4:1 v/v); ii: DCM/TFA (3:1 v/v) and TIS (0.25 eq.); iii: HCOH, or $(CH_3)_2CH_2CHO$, or 4-Cl-PhCHO (1.5 eq.), or 4-F-PhCHO (1.5 eq.), TFA (1.5 eq.), MeOH, MW 45 min at 110 °C; iv: THF, Triphosgene (0.4 eq.), TEA to pH 8.0, RT for 10 min and 4-CH_3O-$BzNH_2$, or 4-CH_3-$BzNH_2$, or $BocNH(CH_2)_3NH_2$ (1.2 eq.) for 1 h.

THBC derivatives **8**, **10**, **13a**, and **15a** were obtained following the procedure described above, by reaction of *L*-Trp-OMe with formaldehyde, isovaleraldehyde, 4-F-benzaldehyde and 4-Cl-benzaldehyde, respectively. Coupling of intermediate **7** with NHBoc-*D*-Phe-OH using HoBt, HBTU in DCM/DMF gave the pseudo dipeptide intermediate **7**. Boc-deprotection of the amino group in TFA acid medium followed by a spontaneous intramolecular cyclization provided the final THBC-based diketopiperazine **8**.

Final THBC-based hydantoin compounds were obtained through the synthetic method reported in Scheme 1. Reaction of the starting THBCs **9a,b**, **11a,b** and **12a b** with triphosgene and different amines, such as 4-OMe, 4-Me-benzylamine, and NHBoc propylendiamine in THF using TEA as base, led to the final hydantoin derivatives **10**, **13a** and **14a** in one step. The reaction of *trans*

(1*R*,3*S*) THBCs **11a** and **12a**, originated the trans derivatives (5*R*,11a*S*) **13a** and **14a**, while the cyclization reaction from the *cis* analogue (1*S*,3*S*) **9b** led to the formation of the trans enantiomer (5*S*,11a*R*) **10.** [1] Removal of the Boc protecting group from **14a** and using TFA and triisopopylsilane (TIS) in dichloromethane led to the final products **15a**.

1.2 Synthesis of 1,3,5-trisubstituted indole derivatives.

The 1,3,5-trisubstituted indole derivatives **22-24** and **26** were obtained as depicted in Scheme 1. Indole-5-carboxaldehyde was reacted with propyl or methyl iodide in DMF using NaH as base to obtain N1-alkylated intermediates **16** and **17** with excellent yields. Both compounds were treated with formaldehyde and commercially available amines in Mannich reaction's conditions, giving the 3-amino alkyl intermediates **18** and **19-21**. Reductive amination of their 5- aldehyde groups with 4-fluoroaniline, using Na(CH3COO)$_3$BH as reducing agents, leads to the hits **22** and **23-25**. Treatment of the intermediate **25** with formaldehyde in the same conditions gave the final N-methylated compound **26**.

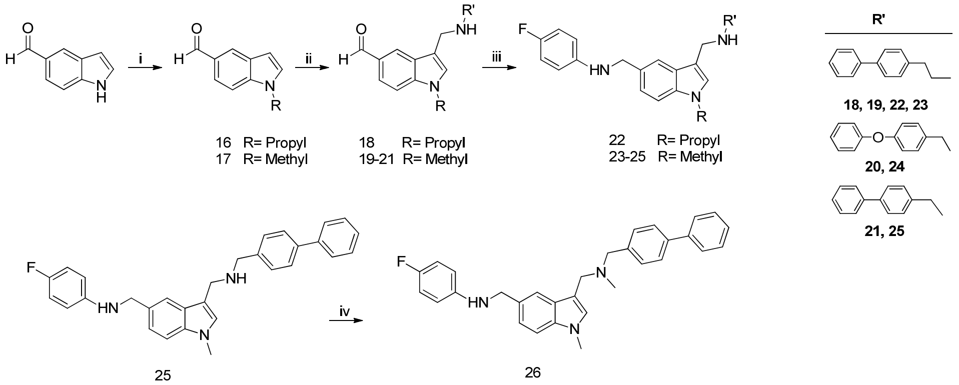

Scheme S2. Synthesis of 1,3,5-trisubstituted indole derivatives. i: alkyliodide (1.5 eq), NaH (1.5 eq), DMF, 0 °C for 30 min then RT for 12 h; ii: CH_2O (2.0 eq), 4-(Ph-Ph)$CH_2CH_2NH_2$ (2.0 eq), or 4-(PhOPh)CH_2NH_2 (2.0 eq), or 4-(Ph-Ph)CH_2NH_2 (2.0 eq), TFA (2.0 eq), DCM, RT, 4-6 h iii: 4-F-$C_6H_4NH_2$ (2.0 eq), CH_2Cl_2/CH_3COOH (5:1, v:v), reflux, 1.5 h then Na(CH3COO)$_3$BH (1.8 eq), reflux, 3-5h; iv: CH2O (1.5 eq), MeOH dry, RT for 1.5 h then NaBH4 (3.0 eq), RT, 3 h

1.3 Synthesis of Synthesis of 5-carboxamide derivatives

N-(4-fluorophenyl)-1-methyl-1H-indole-5-carboxamide (**29**) and N-(4-methyphenyl)-1-methyl-1H-indole-5-carboxamide derivatives **(31**, **32**) were synthesized according to Scheme 3. First, N-1 methylation of indole-5-carboxylic acid and coupling with 4-fuorophenylaniline or 4-methylphenylaniline yielded the 5-carboxamide intermediates **27**, **28**. Mannich reaction of **27** and **28** with formaldehyde and two different secondary amines led to final derivative **29** and the secondary amine intermediate **30**, which was then N-acetylated with acetyl chloride or N-methylated as above described to obtain the final compounds **31** and **32,** respectively.

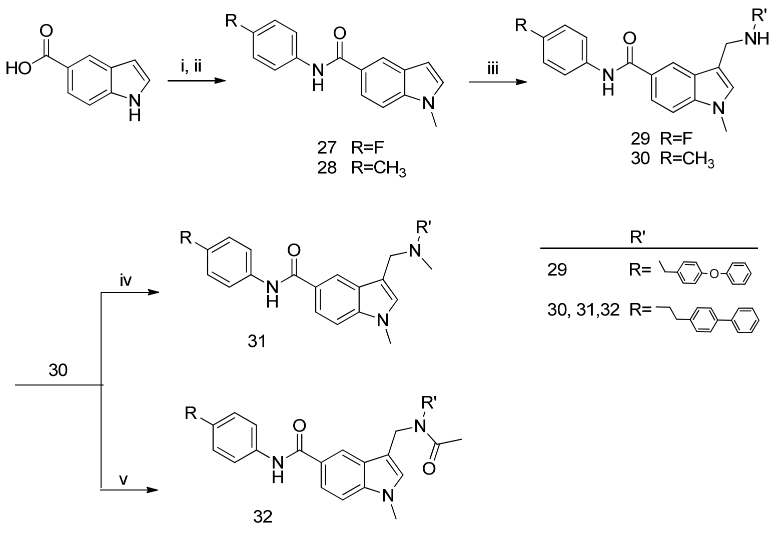

Scheme S3. Synthesis of Synthesis of 5-carboxamide derivatives. i: CH_3I (1.5 eq), NaH (1.5 eq), DMF, 0 °C for 30 min then RT for 12 h; ii: 4-F-$C_6H_4NH_2$ (1.2 eq), PyBop (1.2 eq), DIPEA (2.4 eq), DCM, RT; 12 h; iii: 4-(Ph-Ph)$CH_2CH_2NH_2$ (2.0 eq), or 4-(PhOPh)CH_2NH_2 (2.0 eq), CH2O (2.0 eq), TFA (2.0 eq), DCM, RT, 4-6 h; iv: CH_2O (1.5 eq), MeOH dry, RT for 1.5 h then $NaBH_4$ (3.0 eq), RT, 3 h; v: CH_3COCl (1.2 mmol), TEA (1.2 mmol), DCM, RT, 1 h

2 General

Reagents, starting materials, and solvents were purchased from Sigma-Aldrich (Milan, Italy) and used as received. Reactions were carried out with magnetic stirring in round-bottomed flasks unless otherwise noted. Purifications were conducted on the Biotage Isolera One flash purification system, using prepacked KP-sil columns, (Biotage, Uppsala, Sweden). Microwave assisted closed vessel reactions were performed in a Biotage Initiator þ reactor, using 10-20 mL vials type and external temperature sensor. Analytical thin layer chromatography (TLC) was performed on precoated glass silica gel plates 60 (F254, 0.25 mm, VWR International). 1D-NMR spectra were recorded with Bruker Avance (400 MHz) spectrometer, at room temperature. Spectra were referenced to residual chloroform (7.24 ppm, 1H; 77.23 ppm, 13C) or methanol (3.31 ppm, 1H; 49.15 ppm, 13C). Chemical shifts are reported in d values (ppm) relative to internal Me_4Si, and J values are reported in hertz (Hz). HR-MS experiments were performed using an LTQOrbitrap-XL-ETD mass spectrometer (Thermo Scientific, Bremen, Germany), using electrospray ionization.

General Procedure A: **Pictet–Spengler Reaction** 1 equiv of *L*-tryptophan methyl ester or (S)-2-amino-N-(4-fluorobenzyl)-3-(1Hindol-3-yl)propanamide (**3**) was dissolved in methanol and added with the proper aldehydes (1.5 equiv) and trifluoroacetic acid (1.5 equiv). The mixture was subjected to a microwave assisted closed vessel reaction for 45 min at 110 °C. [2] The mixture was then evaporated in vacuo, and the residue was dissolved in dichloromethane and was

washed three times with water. The organic phase was extracted, dried over Na_2SO_4, filtered, and concentrated under vacuum. The crude products were purified by flash chromatography using mixtures of n-hexane/ethyl acetate as mobile phase.

General Procedure B: Hydantoin Synthesis. Diastereoisomerically pure tetrahydro-β-carbolines (0.2 equiv) were dissolved in THF, and 0.4 equiv of triphosgene was added. The pH was adjusted to 8 by addition of TEA, and the mixture was stirred at room temperature for 10 min. Then, the proper amine (1.2 equiv) was added and the resulting mixture was refluxed for 1 h. After cooling to room temperature, the solvent was evaporated, the residue reconstituted in dichloromethane and washed with water (3 times). The organic phase was extracted, dried over Na_2SO_4, filtered, and concentrated using mixtures of n-hexane/ethyl acetate as mobile phase.

General Procedure C: Boc Removal. The N-Boc protected intermediate (0.2 equiv) was dissolved in a mixture of TFA/DCM (1/3, v/v), and triisopropylsilane (TIS, 0.25 equiv) was added. NaOH (2 N) was added dropwise until pH 7. The mixture was diluted with water and dichloromethane, and the organic phase was extracted, dried over Na_2SO_4, filtered, and concentrated under vacuum. The crude products were purified by flash chromatography using mixtures of n-hexane/ethyl acetate as the mobile phase.

General Procedure D: Coupling reactions.

To a solution of proper carboxylic acids (1.0 equiv) in dichloromethane, different amines (1.2 equiv), PyBop (1.2 equiv) and DIPEA (2.4 equiv) were successively added. Stirring was continued at room temperature for 12 h. Afterward, the reaction mixture was diluted with dichloromethane (20 mL), and the resulting solution was washed successively with 10% citric acid (2 x 25 mL),10% $NaHCO_3$ (2 x 25 mL), and water (2 x 25 mL), dried over Na_2SO_4 and evaporated to dryness. Flash chromatography of the residues, using a mixture of ethyl acetate/n-hexane as eluent systems, yielded, in each case, the corresponding 5-carboxamide indole derivatives.

General Procedure E: Alkylation Reaction.

Indole-5-carboxaldehyde or indole-5-carboxylic acid (1.0 equiv) was dissolved in anhydrous DMF under magnetic stirring, and the temperature was set to 0 °C. To this solution, 1.5 equiv of NaH was added portion wise and the mixture was allowed to react for 30 min. Then, 1.5 equiv of alkyl iodide (methyl iodide or propyl iodide), in DMF was added dropwise and the reaction was warmed to room temperature and maintained under stirring for further 12 h. Then, reaction was quenched by 10% aqueous solution of citric acid and washed with brine. Organic layer was separated, dried over anhydrous Na_2SO_4, filtered, and

evaporated in vacuo. Crude products were purified by column chromatography using n-hexane/ethyl acetate (4:1 v:v) as mobile phase.

General procedure F: Reductive amination reaction

1-alkyl-3-aminomethyl-5-carboxyaldehyde indole intermediates (**18-21)** or **26** or 30 (1.0 equiv) were dissolved in a solution of DCM/CH_3COOH (5:1 v/v) at room temperature. To each solution, an amount of 2.0 equiv of proper amine was added and the mixture was warmed to reflux for 1.5 h. Then, an amount of 1.8 equiv of sodium triacetoxyborohydride was added portion wise and the mixture was allowed to reflux for further 3-5 h. After cooling to room temperature, NaOH 1 N was added. The organic phase was separated and extracted one more time with the alkaline solution. Then, it was dried over Na_2SO_4, filtered, and concentrated in vacuo. The crude products were purified by column chromatography using mixtures of DCM/MeOH as eluent.

General procedure G: Mannick reaction

Formaldehyde (2.0 equiv), trifluoroacetic acid (2.0 equiv) and different commercially available amine (2.0 equiv) were dissolved in dichloromethane and the solution was stirred at room temperature for 30 min. Then, a solution in dichloromethane of the opportune N1 -alkylated indole derivatives (**16, 17**) or of the opportune 1 ,5-disubstituted indole derivatives (**27** and **28**) (1.0 equiv) was added and the mixture was stirring for 4-6 h, following the course of reaction by TLC. When the starting compound disappeared, the reaction was quenched by 10% aqueous solution of sodium bicarbonate and washed with brine, dried over anhydrous Na_2SO_4 and filtered. Organic phase was evaporated in vacuum and 3-aminomethyl indole derivatives were obtained after flash chromatography using a mixture of dichloromethane/methanol (95/5 v:v) as eluent.

General procedure H: Aminomethylation reaction

Compounds 25 or 31 (1.0 equiv) were dissolved in dry methanol under nitrogen atmosphere and molecular sieves were added. A 37% aqueous solution of formaldehyde (1.5 equiv) was introduced after 10 min and the reaction was stirred at room temperature for 1.5 h. Then, $NaBH_4$ (3.0 equiv) was added and the mixture was reacted for further 3 h. Subsequently, the reaction was quenched by 10% aqueous solution of citric acid and the organic solvent was evaporated. Then, dichloromethane was added and the aqueous solution was extracted for three times. The organic layer was dried over Na_2SO_4, filtered, and concentrated in vacuum. The crude products were purified by column chromatography using mixtures of DCM/MeOH as eluent.

(*S*)-tert-butyl (1-((4-fluorobenzyl)amino)-3-(1H-indol-3-yl)-1-oxopropan-2-yl)carbamate (**2**)

Derivative **2** was synthesized starting from NBoc-*L*Trp-OH following procedure D. FC acetate/*n*-hexane 6/4. ^{1}H and DEPT NMR spectra are in accordance with the literature.[1] ESI-MS m/z calcd for $C_{23}H_{26}FN_3O_3$ [(M + H)]+: 411,1958; found 411.1961.

(*S*)-2-amino-N-(4-fluorobenzyl)-3-(1H-indol-3-yl)propanamide (**3**)

Derivative **3** was synthesized starting from **2** following procedure C. FC in ethyl acetate. ^{1}H and DEPT NMR spectra are in accordance with the literature.[1] ESI-MS m/z calcd for $C_{18}H_{18}FN_3O_3$ [(M + H)]+: 312,1507; found 311.1518.

(1S,3S)-N-(4-Fluorobenzyl)-1-isobutyl-2,3,4,9-tetrahydro-1H-pyrido[3,4-b]indole-3-carboxamide (4)

Derivative **4** was synthesized starting from **3** following procedure A. FC in *n*-hexane/ethyl acetate 6/4. ^{1}H and DEPT NMR spectra are in accordance with the literature.[1] ESI-MS *m/z* calcd for $C_{23}H_{26}FN_3O$ $[(M + H)]^+$: 380.2133; found 380.2140.

Methyl 3-((1*S*,3*S*)-3-((4-Fluorobenzyl)carbamoyl)-2,3,4,9- tetrahydro-1H-pyrido[3,4-b]indol-1-yl)propanoate (5)

Derivative **5** was synthesized starting from **3** following procedure A. FC in dichloromethane/methanol 9.5/0.5. ^{1}H and DEPT NMR spectra are in accordance with the literature.[1] ESI-MS *m/z* calcd for $C_{23}H_{24}FN_3O$ $[(M + H)]^+$: 410.1874; found 410.1872.

(*S*)-Methyl 2,3,4,9-Tetrahydro-1H-pyrido[3,4-b]indole-3-carboxylate (6)

Derivative **6** was synthesized starting from *L*-tryptophan methyl ester following procedure A. The product was isolated by filtration from the reaction mixture. ^{1}H and DEPT NMR spectra are in accordance with the literature.[1] ESI-MS m/z calcd for $C_{13}H_{14}N_2O_2$ [(M + H)]+: 230.1055; found 230.2625.

(*S*)-**Methyl 2-((R)-2-((tert-Butoxycarbonyl)amino)-3-phenylpropanoyl)-2,3,4,9-tetrahydro-1H-pyrido[3,4-b]indole-3-carboxylate (7)**

Derivative **7** was synthesized starting from **6** following procedure D. FC in ethyl acetate/*n*-hexane 4/6. ^{1}H and DEPT NMR spectra are in accordance with the literature.[1] ESI-MS m/z calcd for $C_{26}H_{29}N_3O_5$ [(M + H)]+: 463.2107; found 463.5256

(3*R*,12a*S*)-**3-Benzyl-2,3,12,12a-tetrahydropyrazino-[1′,2′:1,6]pyrido[3,4-b]indole-1,4(6H,7H)-dione (8)**

Derivative **8** was synthesized starting from **7** following procedure C. FC in ethyl acetate. ^{1}H and DEPT NMR spectra are in accordance with the literature.[1] ESI-MS m/z calcd for $C_{21}H_{19}N_3O_2$ [(M + H)]+: 346.1550; found 346.1546

(1*R*,3*S*)-**Methyl 1-iIsobutyl-2,3,4,9-tetrahydro-1H-pyrido-[3,4-b]indole-3-carboxylate (9a)** Derivative **9a** was synthesized starting from *L*-tryptophan methyl ester following procedure A. FC in *n*-hexane/ethyl acetate 5/5. Spectral data were in accordance with literature.[1] ESI-MS m/z calcd for $C_{21}H_{19}N_3O_2$ [(M + H)]+: 346.1550; found 346.1546

(1*S*,3*S*)-**Methyl 1-Isobutyl-2,3,4,9-tetrahydro-1H-pyrido[3,4-b]indole-3-carboxylate (9b).** Derivative **9b** was synthesized starting from *L*-tryptophan methyl ester following procedure A. FC in *n*-hexane/ethyl acetate 5/5. Spectral data were in accordance with literature.[1]

(5*S*,11a*R*)-**5-Isobutyl-2-(4-methoxybenzyl)-5,6,11,11a-tetrahydro-1H-imidazo[1′,5:1,6]pyrido[3,4-b]indole-1,3(2H)-dione (10).**

Derivative **10** was synthesized starting from **9b** following procedure B. FC in *n*-hexane/ethyl acetate 5/5. ^{1}H and DEPT NMR spectra are in accordance with the literature.[1] ESI-MS m/z calcd for $C_{25}H_{27}N_3O_3$ [(M + H)]+: 418.2125; found 418.2135

(1*R*,3*S*)**-Methyl 1-(4-Chlorophenyl)-2,3,4,9-tetrahydro-1Hpyrido[3,4-b]indole-3-carboxylate (11a)**

Derivative **11a** was synthesized starting from *L*-tryptophan methyl ester following procedure A. FC in *n*-hexane/ethyl acetate 5/5. Spectral data were in accordance with literature.[1] ESI-MS m/z calcd for $C_{19}H_{17}ClN_2O_2$ [(M + H)]+: 340.0979; found 340.0984

(1*R*,3*S*)-**Methyl 1-(4-Fluorophenyl)-2,3,4,9-tetrahydro-1Hpyrido[3,4-b]indole-3-carboxylate (12a)**

Derivative **12a** was synthesized starting from *L*-tryptophan methyl ester following procedure A. FC in ethyl acetate/*n*-hexane 1/2. Spectral data were in accordance with literature.[1] ESI-MS m/z calcd for $C_{19}H_{17}FN_2O_2$ [(M + H)]+: 324.1274; found 324.1279

(5*R*,11a*S*)**-5-(4-Chlorophenyl)-2-(4-methylbenzyl)-5,6,11,11a-tetrahydro-1H-imidazo[1′,5′:1,6]pyrido[3,4-b]-indole-1,3(2H)-dione (13a)**

Derivative **13a** was synthesized starting from **11a** following procedure B. FC in FC in dichloromethane/ethyl acetate 9.8/0.2. ^{1}H and DEPT NMR spectra are in accordance with the literature.[1] ESI-MS m/z calcd for $C_{27}H_{22}C_1N_3O_2$ [(M + H)]+: 456.1473; found 456.1479

***tert*-Butyl-(3-((5*R*,11a*S*)-5-(4-Fluorophenyl)-1,3-dioxo-11,11a-dihydro-1H-imidazo[1′,5′:1,6]pyrido[3,4-b]indol-2-(3H,5H,6H)-yl)propyl)carbamate (14a)**

Derivative **14a** was synthesized starting from **12a** following procedure B. FC in FC in *n*-hexane/ethyl acetate 2/1. ^{1}H and DEPT NMR spectra are in accordance with the literature.[1] ESI-MS m/z calcd for $C_{27}H_{29}FN_4O_4$ [(M + H)]+: 493.2246; found 493.2250

(5*R*,11a*S*)-2-(3-Aminopropyl)-5-(4-fluorophenyl)-5,6,11,11atetrahydro-1H-imidazo[1′,5′:1,6]pyrido[3,4-b]indole-1,3(2H)-dione (15a)

Derivative **15a** was synthesized starting from **14a** following procedure C. FC in dichloromethane/methanol 9/1. ^{1}H and DEPT NMR spectra are in accordance with the literature.[1] ESI-MS m/z calcd for $C_2H_{21}FN_4O_2$ [(M + H)]+: 93.1721; found 93.1730

1-Propyl-1H-indole-5-carbaldehyde (16)

Derivative **16** was synthesized starting from indole-5-carboxaldehyde following procedure E. FC in *n*-hexane/ethyl acetate 8/2. ^{1}H and DEPT NMR spectra are in accordance with the literature.[3] ESI-MS m/z calcd for $C_{12}H_{14}NO$ [(M + H)]+: 188.1070; found 188.1074

1-Methyl-1H-indole-5-carbaldehyde (17)

Derivative **17** was synthesized starting from indole-5-carboxaldehyde following procedure E. FC in *n*-hexane/ethyl acetate 8/2. ^{1}H and DEPT NMR spectra are in accordance with the literature.[3] ESI-MS m/z calcd for $C_{10}H_{10}NO$ [(M + H)]+: 160.0757; found 160.0765

3-(((2-([1,10-biphenyl]-4-yl)ethyl)amino)methyl)-1-propyl-1H-indole-5-carbaldehyde (18)

Derivative **18** was synthesized starting from **16** following procedure G. FC in dichloromethane/methanol 9.5/0.5. ^{1}H and DEPT NMR spectra are in accordance with the literature.[3] ESI-MS m/z calcd for $C_{27}H_{29}N_{2}O$ [(M + H)]+: 397.2274; found 397.2282

3-(((2-([1,10-biphenyl]-4-yl)ethyl)amino)methyl)-1-methyl-1H-indole-5-carbaldehyde (19)

Derivative **19** was synthesized starting from **17** following procedure G. FC in dichloromethane/methanol 9.5/0.5. ^{1}H and DEPT NMR spectra are in accordance with the literature.[3] ESI-MS m/z calcd for $C_{25}H_{25}N_{2}O$ [(M + H)]+: 369.1961; found 369.1966,

1-Methyl-3-(((4-phenoxybenzyl)amino)methyl)-1H-indole-5-carbaldehyde (20)

Derivative **20** was synthesized starting from **17** following procedure G. FC in dichloromethane/methanol 9.5/0.5. ^{1}H and DEPT NMR spectra are in accordance with the literature.[3] ESI-MS m/z calcd for $C_{24}H_{23}N_{2}O_{2}$ [(M + H)]+: 371.1754; found 371.1758,

3-((([1,10-biphenyl]-4-ylmethyl)amino)methyl)-1-methyl-1H-indole-5-carbaldehyde (21)

Derivative **21** was synthesized starting from **17** following procedure G. FC in dichloromethane/methanol 9.5/0.5. ^{1}H and DEPT NMR spectra are in accordance with the literature.[3] ESI-MS m/z calcd for $C_{24}H_{23}N_{2}O$ [(M + H)]+: 355.1805; found 355.1812

N-((3-(((2-([1,10-biphenyl]-4-yl)ethyl)amino)methyl)-1-propyl-1H-indol-5-yl)methyl)-4-fluoroaniline (22)

Derivative **22** was synthesized starting from **18** following procedure F. FC in dichloromethane/methanol 9.8/0.2. ^{1}H and DEPT NMR spectra are in accordance with the literature.[3] ESI-MS m/z calcd for $C_{33}H_{35}FN_{3}$ [(M + H)]+: 492.2814,; found 492.2819

N-((3-(((2-([1,10-biphenyl]-4-yl)ethyl)amino)methyl)-1-methyl-1H-indol-5-yl)methyl)-4-fluoroaniline (23)

Derivative **23** was synthesized starting from **19** following procedure F. FC in dichloromethane/methanol 9.8/0.2. ^{1}H and DEPT NMR spectra are in accordance with the literature.[3] ESI-MS m/z calcd for $C_{31}H_{31}FN_3$ [(M + H)]+: 464.2497; found 464.2513

4-Fluoro-N-((1-methyl-3-(((4-phenoxybenzyl)amino)methyl)-1H-indol-5-yl)methyl)aniline (24)

Derivative **24** was synthesized starting from **20** following procedure F. FC in dichloromethane/methanol 9.8/0.2. ^{1}H and DEPT NMR spectra are in accordance with the literature.[3] ESI-MS m/z calcd for $C_{30}H_{29}FN_3O$ [(M + H)]+: 466.2289; found 466.2292

N-((3-((([1,10-biphenyl]-4-ylmethyl)amino)methyl)-1-methyl-1H-indol-5-yl)methyl)-4-fluoroaniline (25)

4-Fluoro-N-((1-methyl-3-(((4-phenoxybenzyl)amino)methyl)-1H-indol-5-yl)methyl)aniline (24)

Derivative **25** was synthesized starting from **21** following procedure F. FC in dichloromethane/methanol 9.8/0.2. ^{1}H and DEPT NMR spectra are in accordance with the literature.[3] ESI-MS m/z calcd for $C_{30}H_{29}FN_3$ [(M + H)]+: 450.2340; found 450.2349

N-((3-((([1,10-biphenyl]-4-ylmethyl)(methyl)amino)methyl)-1-methyl-1H-indol-5-yl)methyl)-4-fluoroaniline (26)

Derivative **26** was synthesized starting from **25** following procedure H. FC in dichloromethane/methanol 9.8/0.2. ^{1}H and DEPT NMR spectra are in accordance with the literature.[3] ESI-MS m/z calcd for $C_{31}H_{21}FN_3$ [(M + H)]+: 464.2497; found 450.2514

N-(4-fluorophenyl)-1-methyl-1H-indole-5-carboxamide (27)

Derivative **27** was synthesized starting from 1-methyl-1H-indole-5-carboxylic acids, previously synthesized following procedure E. Then, compound **27** was finally obtained following procedure D. FC in acetate/*n*-hexane 8/2. ^{1}H and DEPT NMR spectra are in accordance with the literature.[3] ESI-MS m/z calcd for $C_{16}H_{14}FN_2O$ [(M + H)]+: 269.1085; found 269.1093

1-Methyl-N-(4-tolyl)-1H-indole-5-carboxamide (28)

Derivative **28** was synthesized starting from 1-methyl-1H-indole-5-carboxylic acids, previously synthesized following procedure E. Then, compound **28** was finally obtained following procedure D. FC in acetate/*n*-hexane 8/2. ^{1}H and DEPT NMR spectra are in accordance with the literature.[3] ESI-MS m/z calcd for $C_{16}H_{14}FN_2O$ [(M + H)]+: 269.1085; found 269.1093

N-(4-fluorophenyl)-1-methyl-3-(((4-phenoxyphenethyl)amino)methyl)-1H-indole-5-carboxamide (29)

Derivative **29** was synthesized starting from **27** following procedure G. FC in dichloromethane/methanol 9.5/0.5. 1H and DEPT NMR spectra are in accordance

with the literature.[3] ESI-MS m/z calcd for $C_{31}H_{29}FN_3O_2$ [(M + H)]+: 494.2238; found 494.2246

3-(((2-([1,10-biphenyl]-4-yl)ethyl)amino)methyl)-1-methyl-N-(p-tolyl)-1H-indole-5-carboxamide (30)

Derivative **30** was synthesized starting from **28** following procedure G. FC in dichloromethane/methanol 9.5/0.5. 1H and DEPT NMR spectra are in accordance with the literature.[3] ESI-MS m/z calcd for $C_{32}H_{32}N_3O$ [(M + H)]+: 474.2540; found 474.2543

3-(((2-([1,10-biphenyl]-4-yl)ethyl)(methyl)amino)methyl)-1-methyl-N-(p-tolyl)-1H-indole-5-carboxamide (31)

Derivative **31** was synthesized starting from **30** following procedure H. FC in dichloromethane/methanol 9.8/0.2. 1H and DEPT NMR spectra are in accordance with the literature.[3] ESI-MS m/z calcd for $C_{33}H_{34}N_3O$ [(M + H)]+: 488.2696; found 488.2702

Synthesis of 3-((N-(2-([1,10-biphenyl]-4-yl)ethyl)acetamido)methyl)-1-methyl-N-(p-tolyl)-1H-indole-5-carboxamide (32)

Intermediate 31 (1.0 equiv) was dissolved in dichloromethane. A solution of acetyl chloride (1.2 equiv) and triethylamine (1.2 equiv) in dichloromethane was added at room temperature. The reaction was stirred for 1 h, then the mixture was washed with brine, the organic phase was dried over Na_2SO_4, filtered, and concentrated in vacuo. The crude products were purified by column chromatography using mixtures of ethyl acetate/n-hexane as eluent leading to the corresponding acetylated derivative **32** as rotamers mixtures. 1H and DEPT NMR spectra are in accordance with the literature.[3] ESI-MS m/z calcd for $C_{34}H_{33}N_3O_2$ [(M + H)]+: 515.2573; found 515.2577

3. Purification and characterization

All final compounds were purified by RP-HPLC on a preparative C18- bonded silica column (Phenomenex Kinetex Biphenyl 100 Å, 100 × 21.2 mm, 5 μ m) using a Shimadzu SPD 20 A UV/VIS detector, with detection at 214 and 254 nm. Mobile phase was: (A) H 2 O and (B) ACN, both acidified with 0.1% TFA (v/v). Injection volume was 5000 9 μ L; flowrate was set to 17 mL/min. The following gradient was employed: 0–18 min, 1–40% B, 18.01–20 min, 40–70% B, 20.01–21 min, 70–90% B, 21.01–23 min, returning to 1% B. Analytical purity and retention time (tr) of each compound were determined using HPLC conditions in the above solvent system (solvents A and B) programmed at a flow rate of 0.600 ml/min, fitted with C-18 column Phenomenex, Kinetex Biphenyl 100 Å C18 column (100x3.00 mm, 2.6 μm). LC gradient was the following: 0–7 min, 1–40% B, 7.01–8 min, 40–90% B, 8.01–9 min, returning to 1% B, 9–11 min, isocratic for 2 min. All analogues showed ≥97% purity when monitored at 220 nm (Figures S1–S13). Ultra-high resolution mass spectra were obtained by positive ESI infusion on a LTQ Orbitrap XL mass spectrometer (Thermo Scientific, Germany), equipped with the Xcalibur software for processing the data acquired. The sample was dissolved in a mixture of water

and methanol (50/50) and injected directly into the electrospray source, using a syringe pump, at constant flow (15 μL/min).

4. Analytical HPLC traces of tested compounds

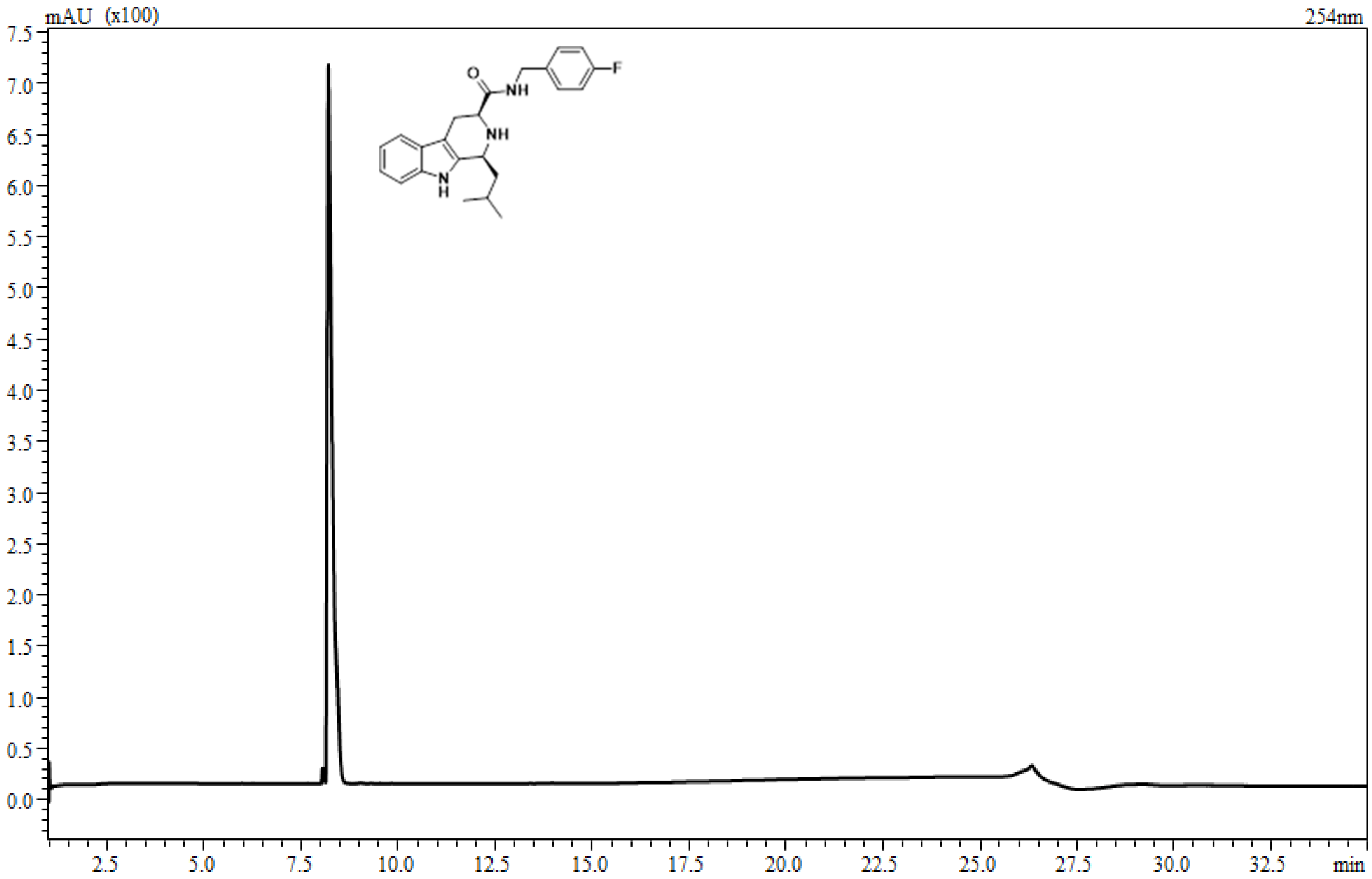

Figure S3. Analytical HPLC trace at 220 nm of compound **4**.

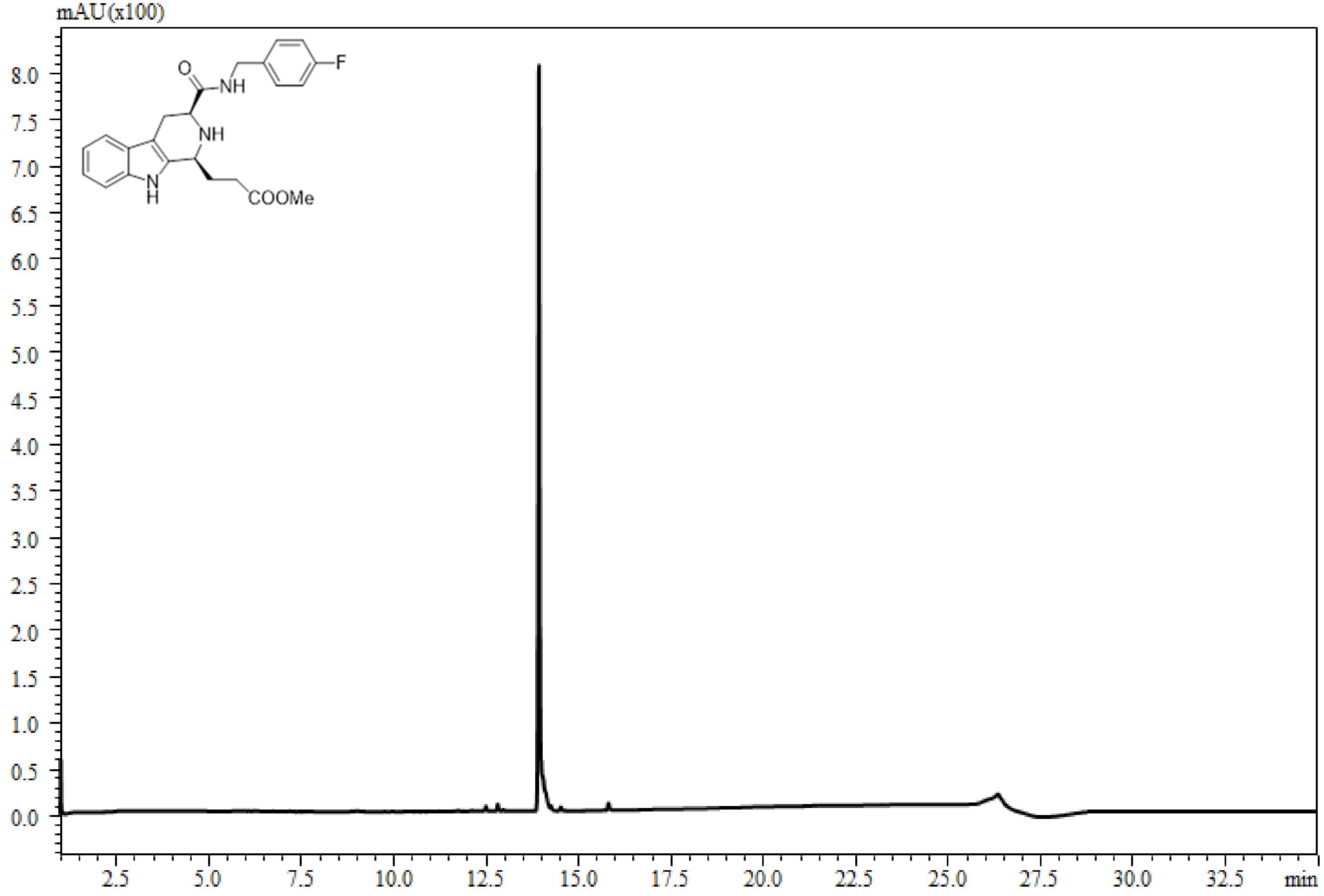

Figure S4. Analytical HPLC trace at 220 nm of compound **5**.

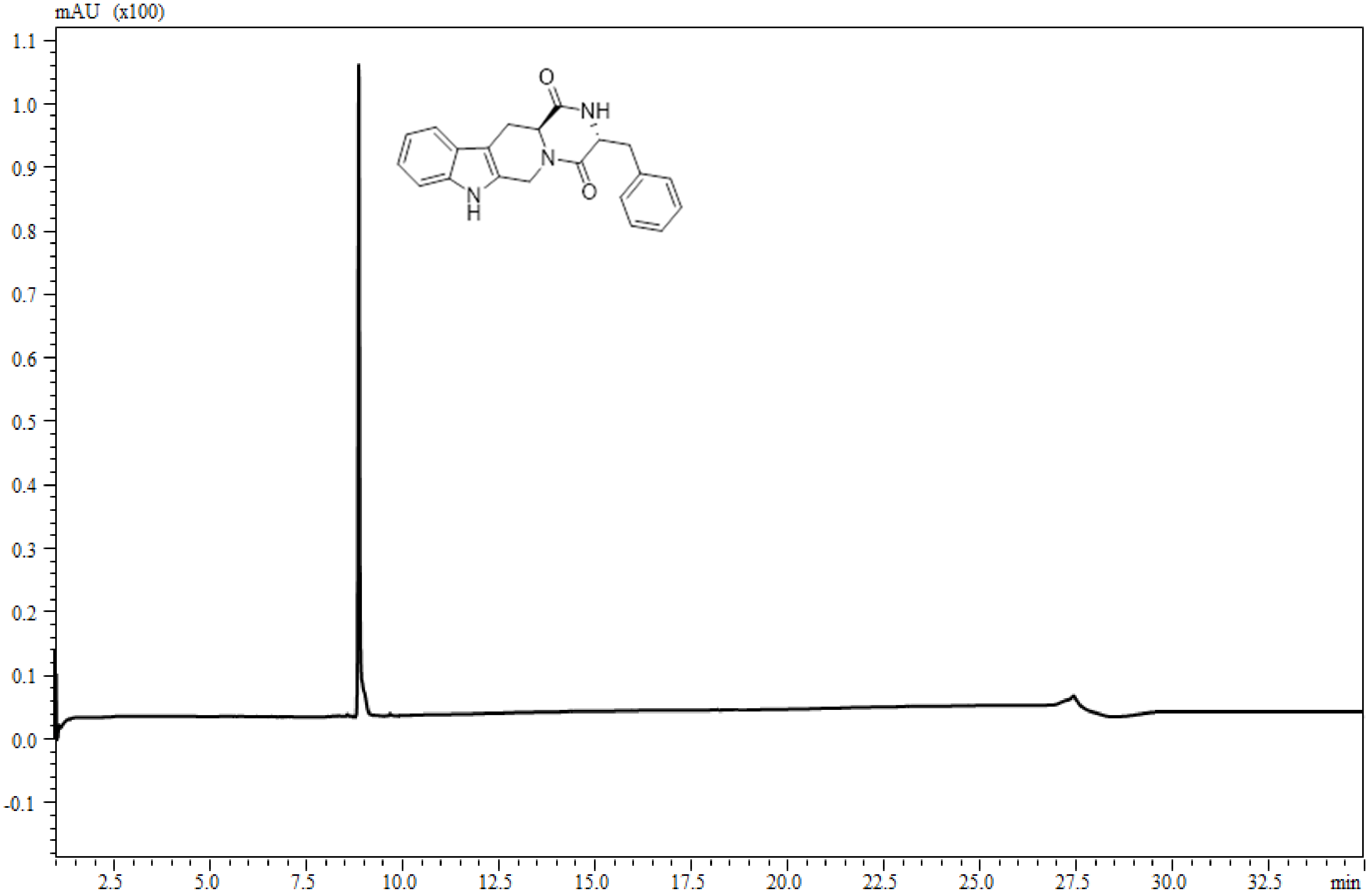

Figure S5. Analytical HPLC trace at 220 nm of compound **8**.

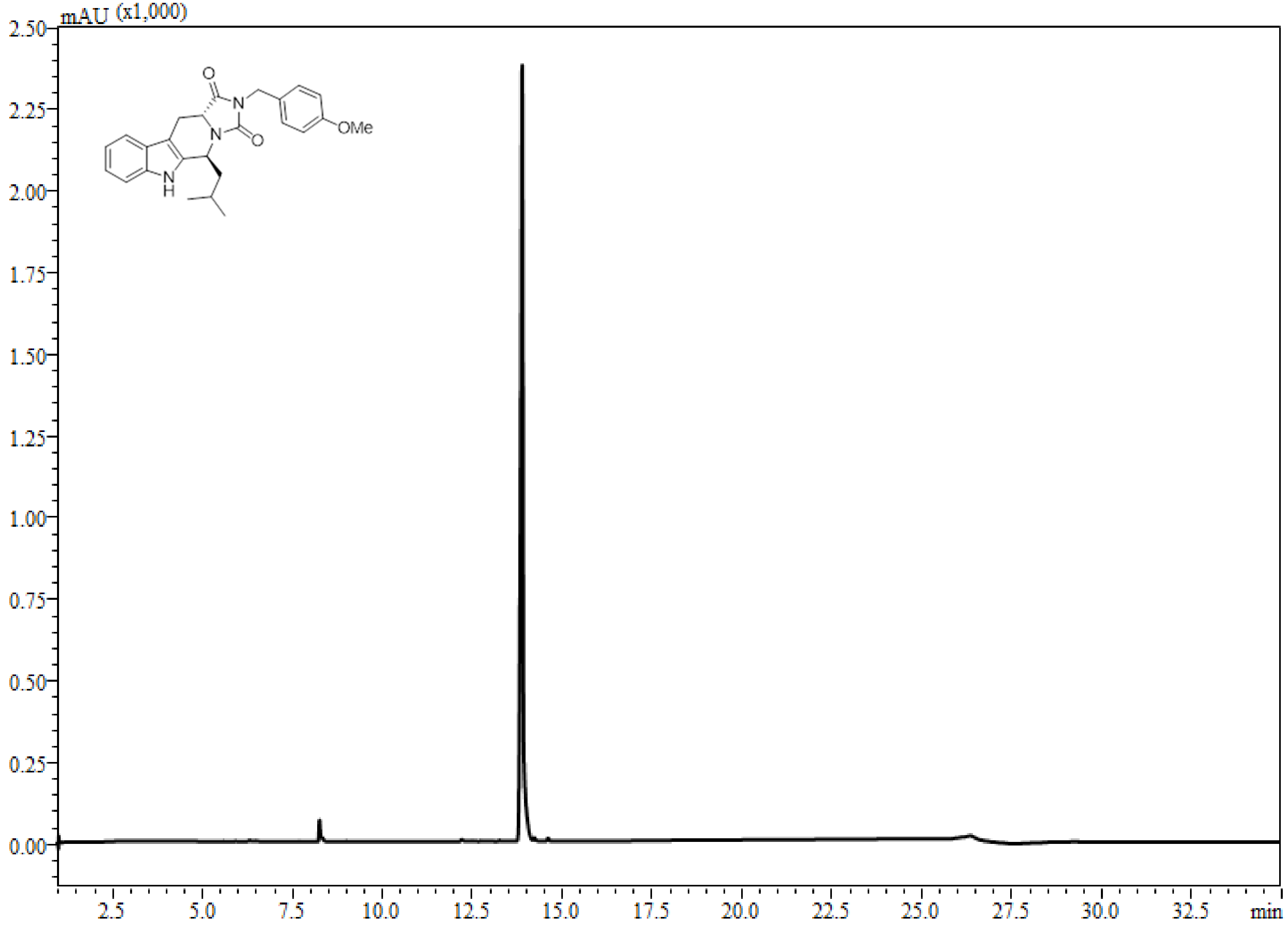

Figure S6. Analytical HPLC trace at 220 nm of compound **10**.

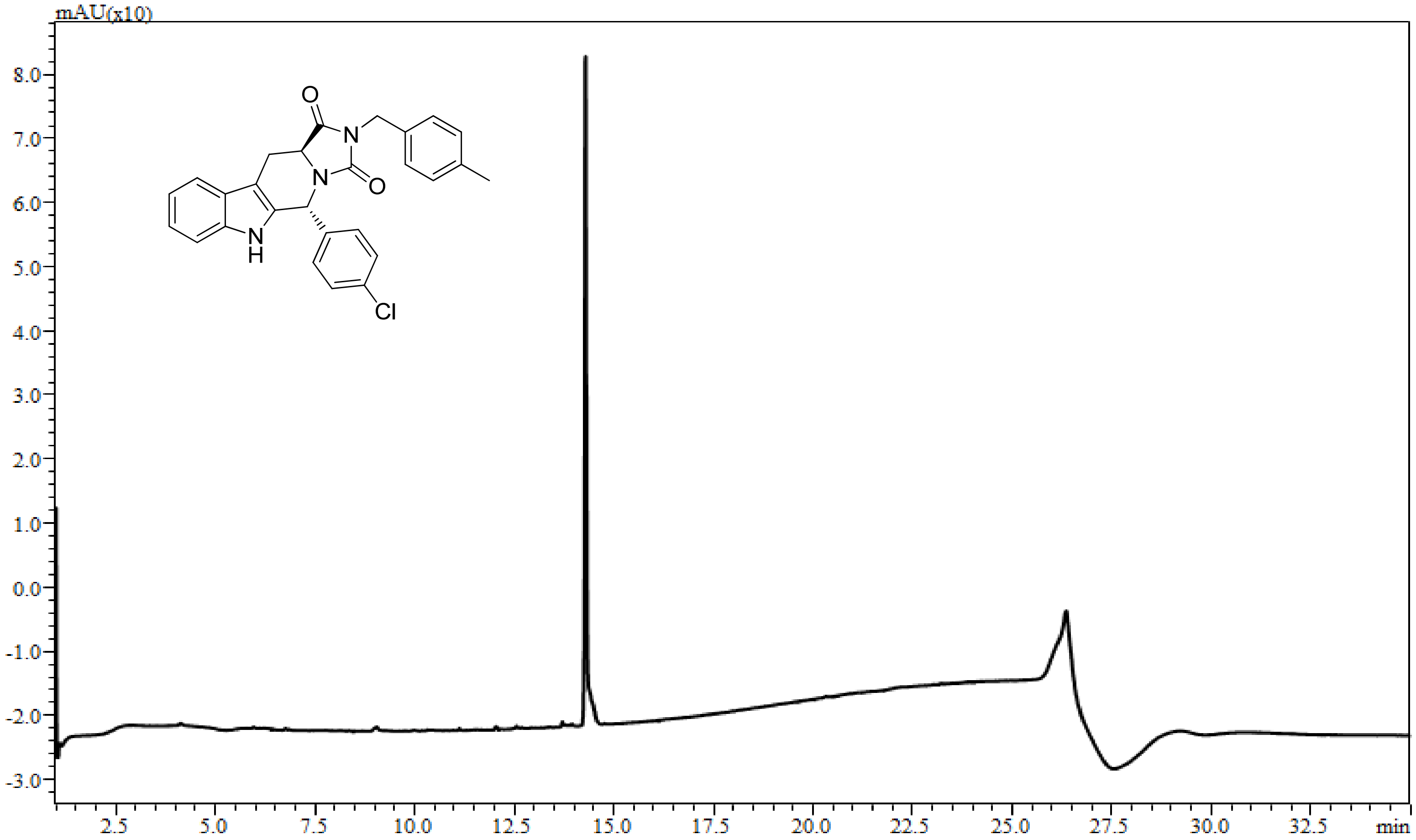

Figure S7. Analytical HPLC trace at 220 nm of compound **13a.**

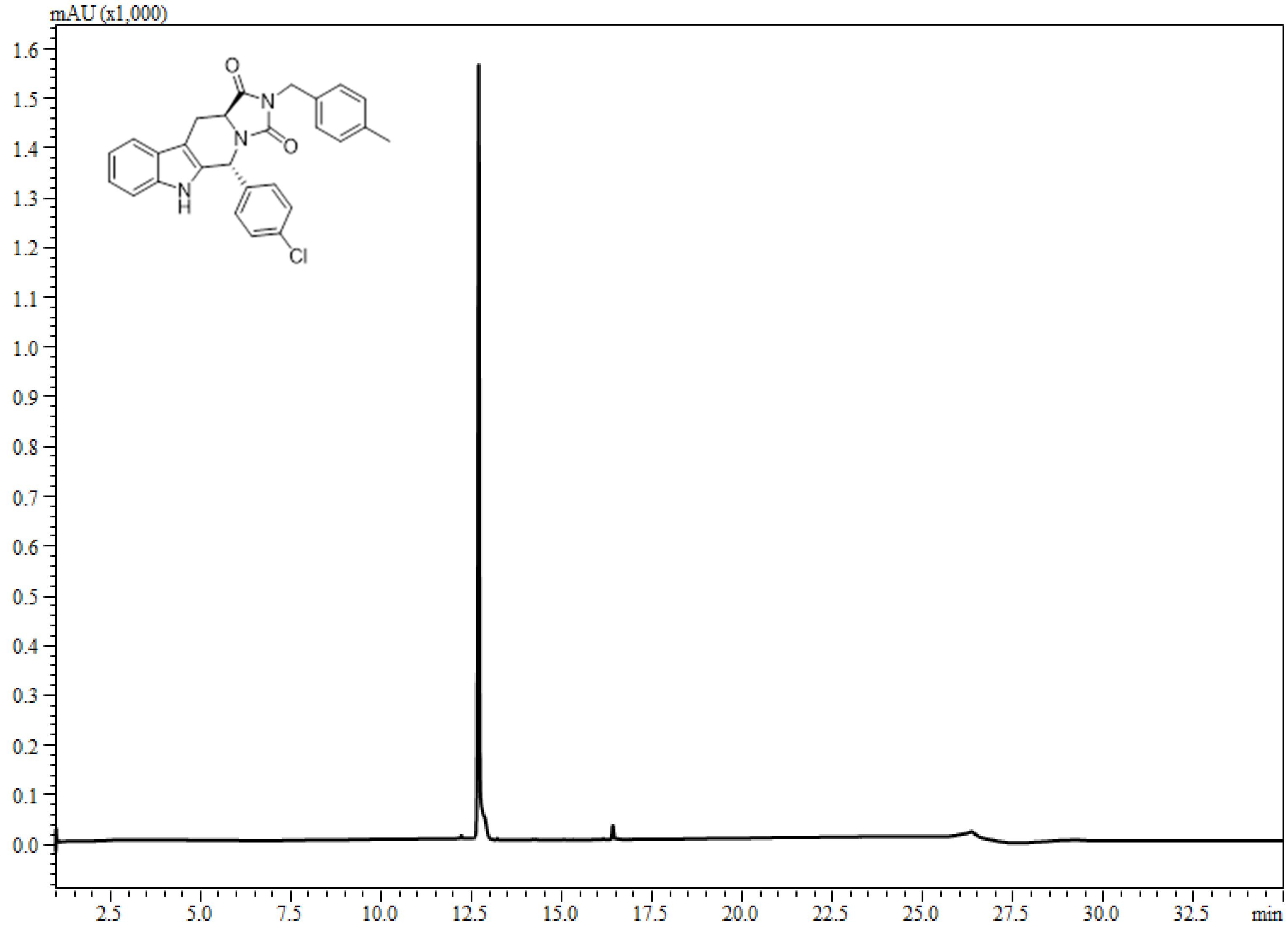

Figure S8. Analytical HPLC trace at 220 nm of compound **15a**.

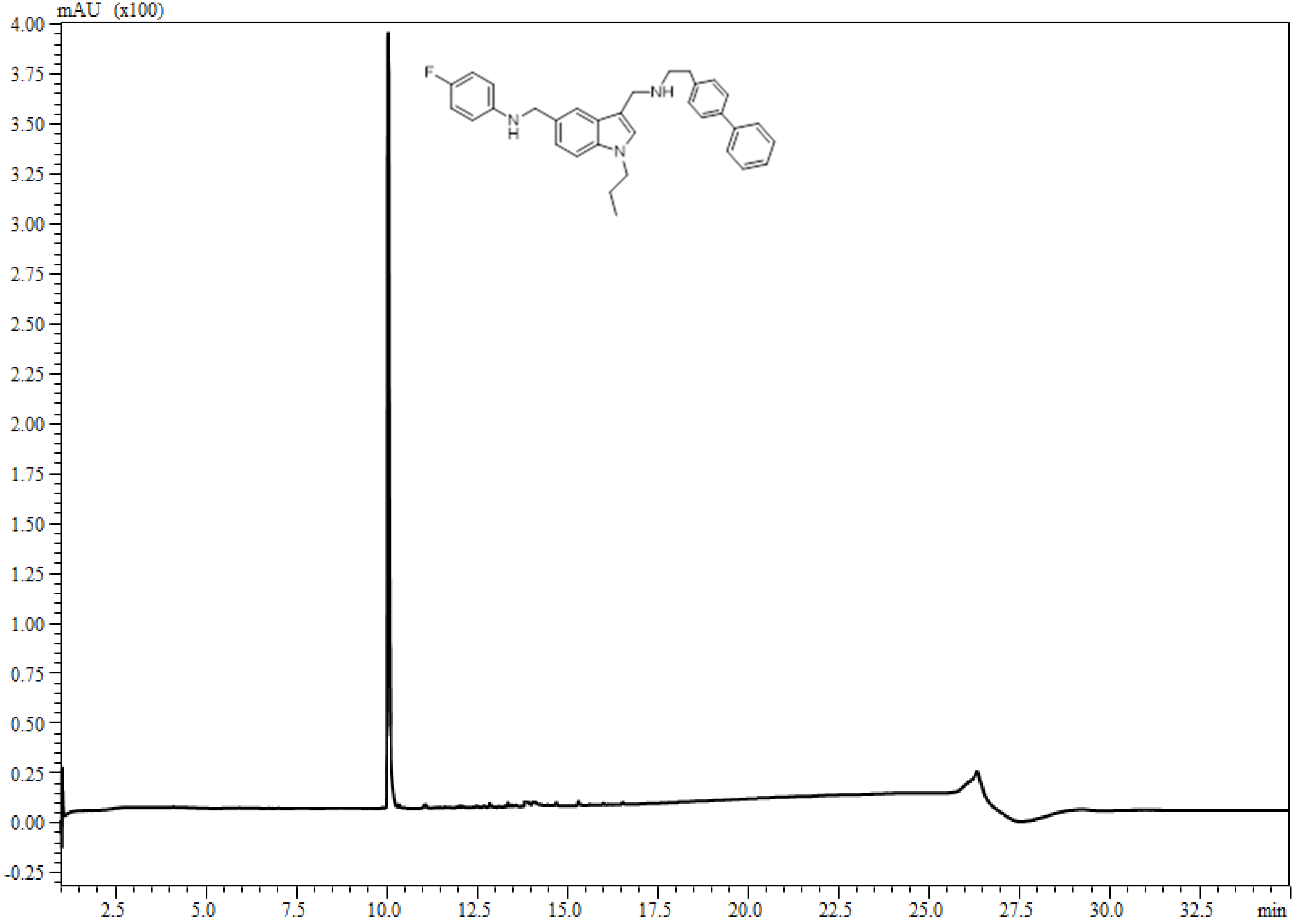

Figure S9. Analytical HPLC trace at 220 nm of compound **22.**

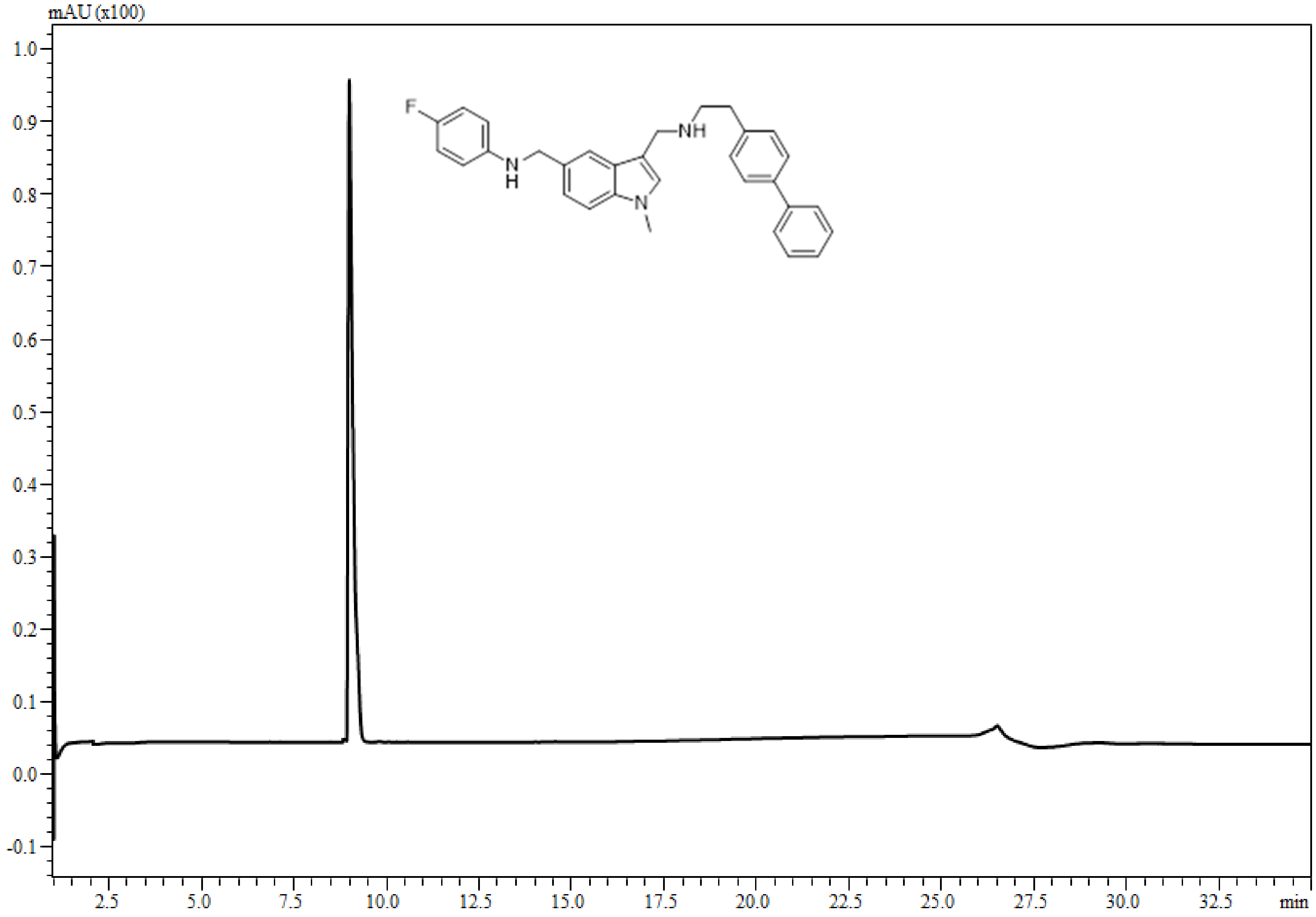

Figure S10. Analytical HPLC trace at 220 nm of compound **23.**

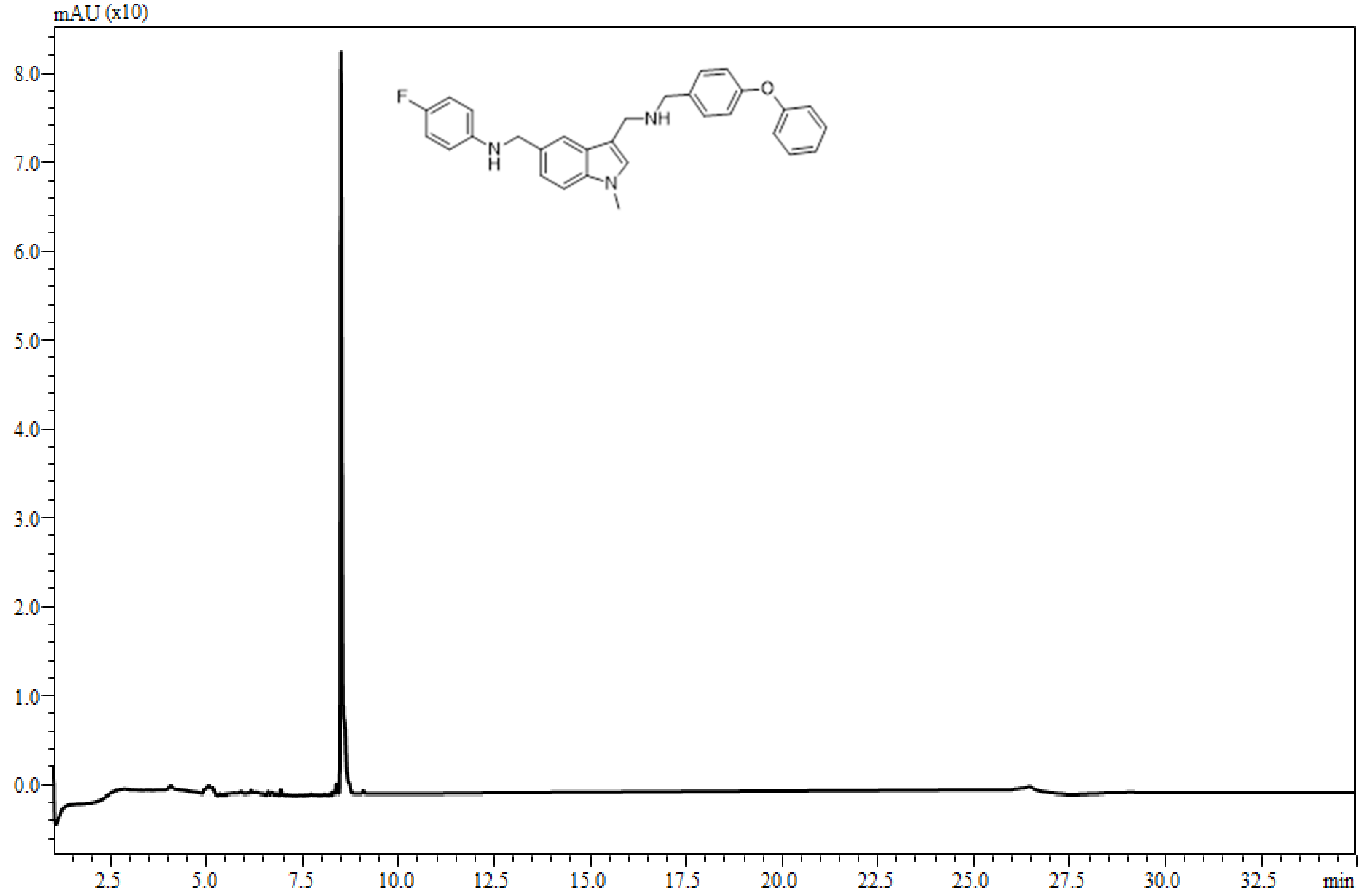

Figure S11. Analytical HPLC trace at 220 nm of compound **24.**

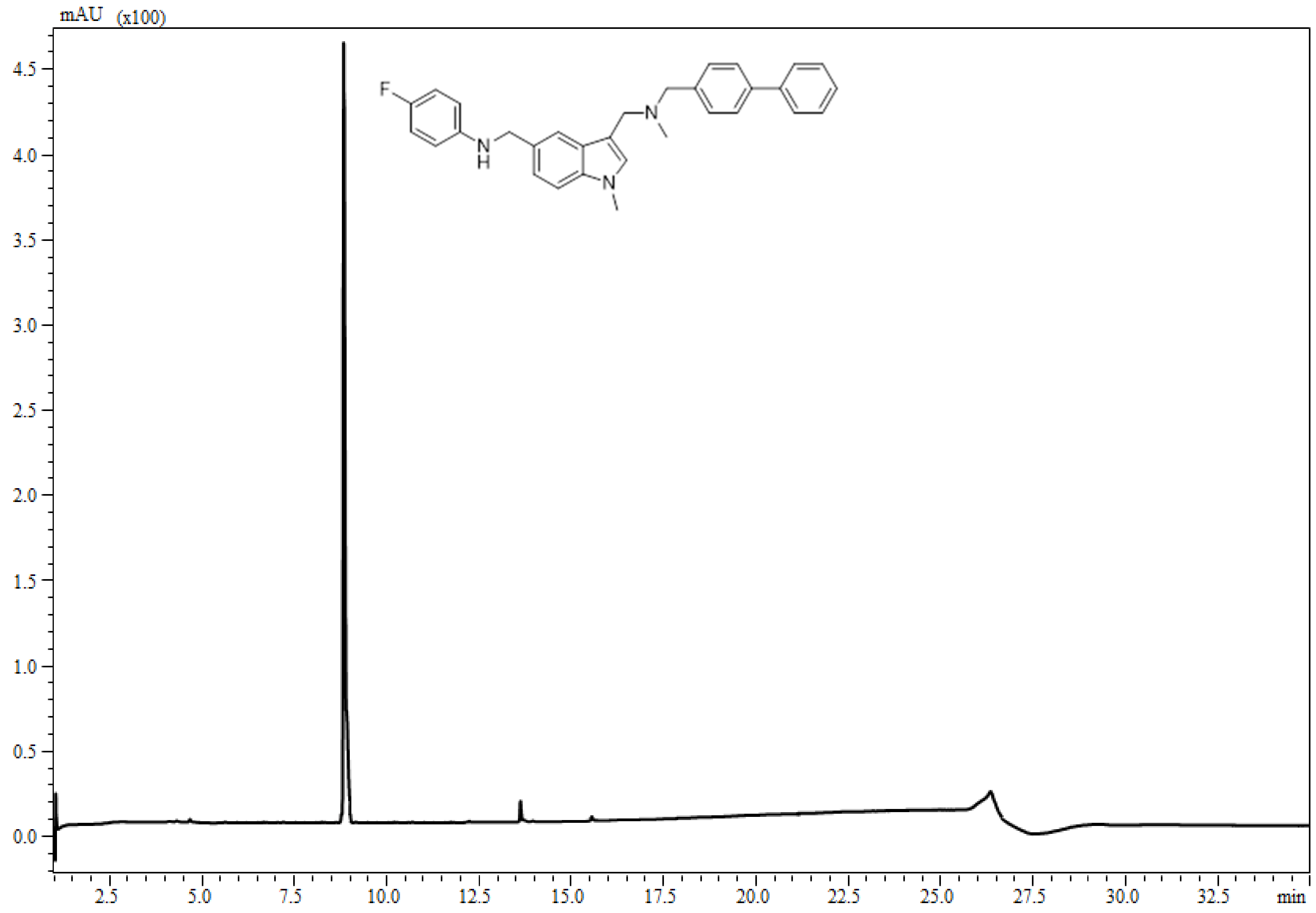

Figure S12. Analytical HPLC trace at 220 nm of compound **26.**

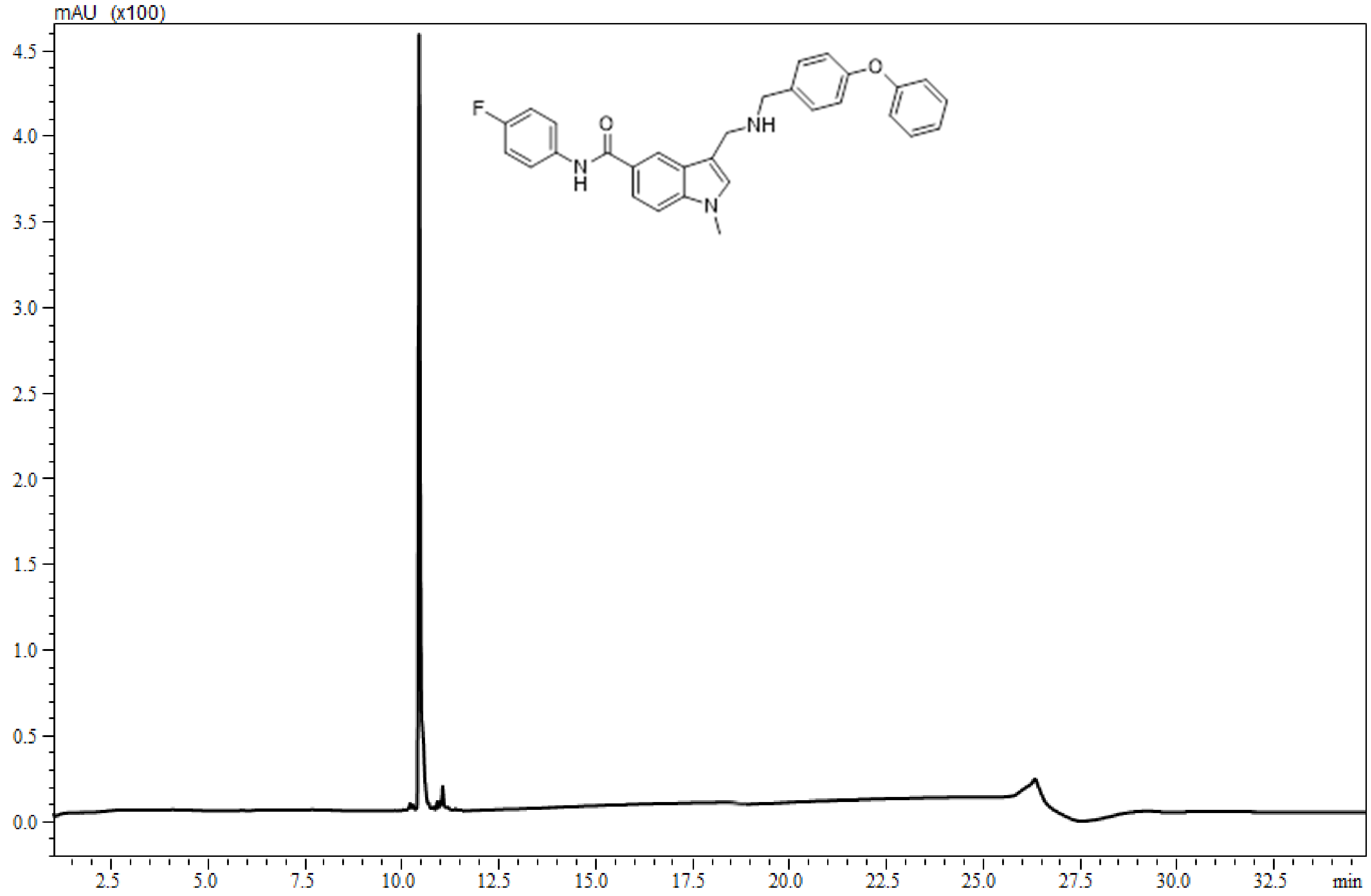

Figure S13. Analytical HPLC trace at 220 nm of compound **29.**

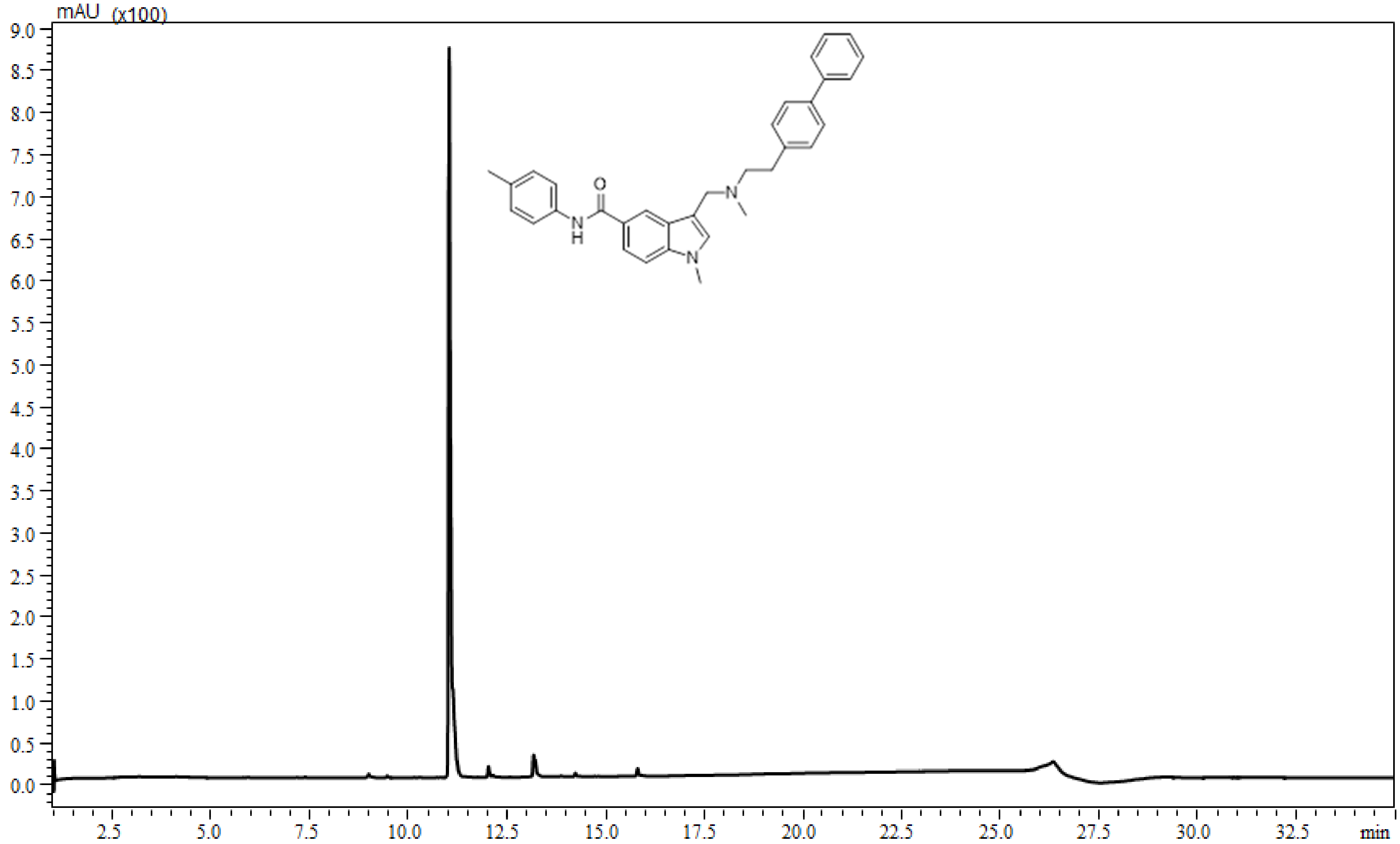

Figure S14. Analytical HPLC trace at 220 nm of compound **31.**

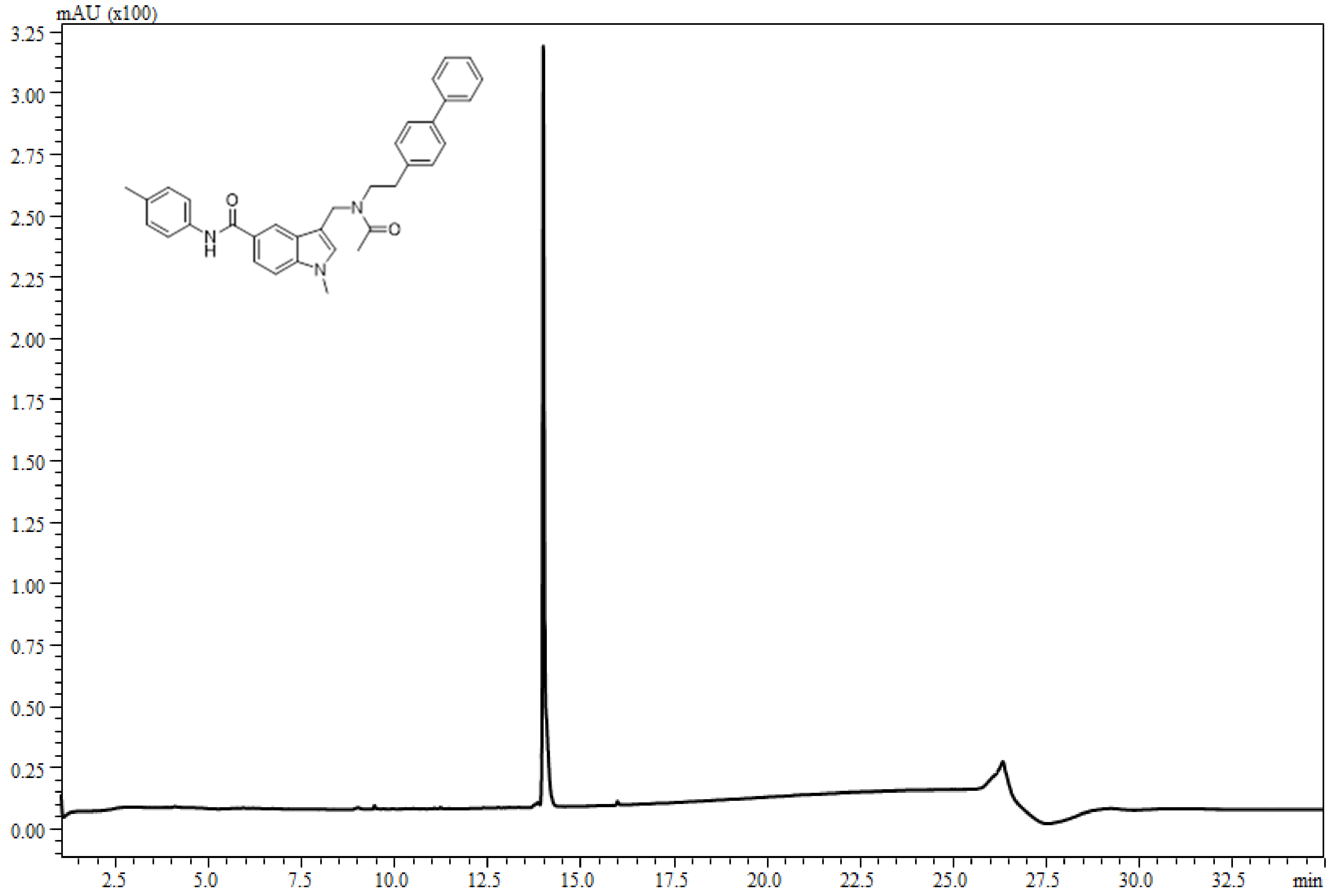

Figure S15. Analytical HPLC trace at 220 nm of compound **32**

References

1. Bertamino, A.; Ostacolo, C.; Medina, A.; Di Sarno, V.; Lauro, G.; Ciaglia, T.; Vestuto, V.; Pepe, G.; Basilicata, M. G.; Musella, S.; Smaldone, G.; Cristiano, C.; Gonzalez-Rodriguez, S.; Fernandez-Carvajal, A.; Bifulco, G.; Campiglia, P.; Gomez-Monterrey, I.; Russo, R., Exploration of TRPM8 Binding Sites by β-Carboline-Based Antagonists and Their In Vitro Characterization and In Vivo Analgesic Activities. *Journal of Medicinal Chemistry* **2020,** 63, (17), 9672-9694.
2. Bertamino, A.; Lauro, G.; Ostacolo, C.; Di Sarno, V.; Musella, S.; Ciaglia, T.; Campiglia, P.; Bifulco, G.; Gomez-Monterrey, I. M., Ring-Fused Cyclic Aminals from Tetrahydro-β-carboline-Based Dipeptide Compounds. *The Journal of Organic Chemistry* **2017,** 82, (23), 12014-12027.
3. Ostacolo, C.; Di Sarno, V.; Lauro, G.; Pepe, G.; Musella, S.; Ciaglia, T.; Vestuto, V.; Autore, G.; Bifulco, G.; Marzocco, S.; Campiglia, P.; Gomez-Monterrey, I. M.; Bertamino, A., Identification of an indol-based multi-target kinase inhibitor through phenotype screening and target fishing using inverse virtual screening approach. *European Journal of Medicinal Chemistry* **2019,** 167, 61-75.
